# Multiple trauma management in mountain environments - a scoping review: Evidence based guidelines of the International Commission for Mountain Emergency Medicine (ICAR MedCom). Intended for physicians and other advanced life support personnel

**DOI:** 10.1186/s13049-020-00790-1

**Published:** 2020-12-14

**Authors:** G. Sumann, D. Moens, B. Brink, M. Brodmann Maeder, M. Greene, M. Jacob, P. Koirala, K. Zafren, M. Ayala, M. Musi, K. Oshiro, A. Sheets, G. Strapazzon, D. Macias, P. Paal

**Affiliations:** 1 Austrian Society of Mountain and High Altitude Medicine, Emergency physician, Austrian Mountain and Helicopter Rescue, Altach, Austria; 2 Emergency Department Liège University Hospital, CMH HEMS Lead physician and medical director, Senior Lecturer at the University of Liège, Liège, Belgium; 3 Mountain Emergency Paramedic, AHEMS, Canadian Society of Mountain Medicine, Whistler Blackcomb Ski Patrol, Whistler, Canada; 4 Department of Emergency Medicine, University Hospital and University of Bern, Switzerland and Institute of Mountain Emergency Medicine, Eurac Research, Bolzano, Italy; 5 Medical Officer Mountain Rescue England and Wales, Wales, UK; 6 Department of Anaesthesiology, Intensive Care and Pain Medicine, Hospitallers Brothers Saint-Elisabeth-Hospital Straubing, Bavarian Mountain Rescue Service, Straubing, Germany; 7 Adjunct Assistant Professor, Emergency Medicine, University of Maryland School of Medicine, Mountain Medicine Society of Nepal, Kathmandu, Nepal; 8 ICAR MedCom, Department of Emergency Medicine, Stanford University Medical Center, Stanford, CA USA; 9 Alaska Native Medical Center, Anchorage, AK USA; 10 University Hospital Germans Trias i Pujol, Badalona, Spain; 11 Department of Emergency Medicine, University of Colorado School of Medicine, Aurora, CO USA; 12 Department of Cardiovascular Medicine and Director of Mountain Medicine, Research, and Survey Division, Hokkaido Ohno Memorial Hospital, Sapporo, Japan; 13 Emergency Department, Boulder Community Health, Boulder, CO USA; 14 Institute of Mountain Emergency Medicine, Eurac Research, Bolzano, Italy; 15 The Corpo Nazionale Soccorso Alpino e Speleologico, National Medical School (CNSAS SNaMed), Milan, Italy; 16 Department of Emergency Medicine, International Mountain Medicine Center, University of New Mexico, Albuquerque, NM USA; 17 Department of Anaesthesiology and Intensive Care Medicine, St. John of God Hospital, Paracelsus Medical University, Salzburg, Austria

**Keywords:** analgesia, Advanced Trauma Life Support, emergency medical services, first aid, haemorrhage, multiple trauma, shock, triage, wounds and injuries

## Abstract

**Background:**

Multiple trauma in mountain environments may be associated with increased morbidity and mortality compared to urban environments.

**Objective:**

To provide evidence based guidance to assist rescuers in multiple trauma management in mountain environments.

**Eligibility criteria:**

All articles published on or before September 30th 2019, in all languages, were included. Articles were searched with predefined search terms.

**Sources of evidence:**

PubMed, Cochrane Database of Systematic Reviews and hand searching of relevant studies from the reference list of included articles.

**Charting methods:**

Evidence was searched according to clinically relevant topics and PICO questions.

**Results:**

Two-hundred forty-seven articles met the inclusion criteria. Recommendations were developed and graded according to the evidence-grading system of the American College of Chest Physicians. The manuscript was initially written and discussed by the coauthors. Then it was presented to ICAR MedCom in draft and again in final form for discussion and internal peer review. Finally, in a face-to-face discussion within ICAR MedCom consensus was reached on October 11th 2019, at the ICAR fall meeting in Zakopane, Poland.

**Conclusions:**

Multiple trauma management in mountain environments can be demanding. Safety of the rescuers and the victim has priority. A crABCDE approach, with haemorrhage control first, is central, followed by basic first aid, splinting, immobilisation, analgesia, and insulation. Time for on-site medical treatment must be balanced against the need for rapid transfer to a trauma centre and should be as short as possible. Reduced on-scene times may be achieved with helicopter rescue. Advanced diagnostics (e.g. ultrasound) may be used and treatment continued during transport.

## Introduction

In mountain environments, multiple trauma, a life threatening injury involving at least one body region with an injury severity score (ISS) ≥16, may be associated with increased prehospital time, a higher risk of accidental hypothermia, and a lower systolic blood pressure compared to urban trauma [1]. In a survey from Scotland, 78.4% of survivors were traumatised (*n* = 622), but only 12 (3.6%) had sustained multiple trauma [2], indicating that multiple trauma is a rare condition. However, a multiple-trauma patients requires more resources. Treatment cost may exceed US$ 1 million [3] and quality of life and capacity to work are often permanently impaired [4]. Outcome from multiple trauma on a mountain may be worse than in an urban environment. It is necessary to optimise prehospital care of multiple trauma patients to avoid poor outcomes related to delayed or incorrect treatment. No specific guidelines exist for the management of multiple trauma in mountain environments. Despite numerous medical and technological advances, care of multiple trauma patients in a mountain environment remains challenging. Bad weather, difficult terrain, poor visibility, and limited rescue personnel and transport options may affect patient outcomes. Every rescue is different. Rescuers must exercise flexibility in selecting the transport options best suited to each case. The objective of this review is to provide evidence based guidance to assist rescuers in the management of multiple trauma in mountain environments.

## Methods

For this PRISMA Scoping Review (PRISMA-ScR) [5], a working group was formed at the ICAR meeting in Soldeu, Andorra in October 2017. Subgroups of coauthors were invited, based on their interests and knowledge, to collaborate under the coordination of a lead author for each subtopic. A PRISMA-ScR checklist is provided (Supplemental Table 1). Population Intervention Comparator Outcome (PICO) questions were developed and evidence mapped according to clinically relevant challenges and PICO questions (Supplemental file 1). All articles published on or before September 30th 2019, in all languages, were included. Searches of PubMed and the Cochrane Database of Systematic Reviews and hand searching of relevant studies from the reference lists of included articles were performed (Supplemental file 2). Recommendations were developed and graded according to the evidence-grading system of the American College of Chest Physicians (Table 1) [6]. The manuscript was written and discussed by the coauthors. It was presented in draft and again in final form for discussion and internal peer review within ICAR MedCom. Finally, in a face-to-face discussion of ICAR MedCom, consensus was reached on October 11th 2019 at the ICAR meeting in Zakopane, Poland.

**Table 1:** Classification scheme for grading evidence [6]

Grade 1A	Strong recommendation, high quality evidence, benefits clearly outweigh risks and burden or vice versa
Grade 1B	Strong recommendation, moderate-quality evidence, benefits clearly outweigh risks and burdens or vice versa
Grade 1C	Strong recommendation, low-quality or very low-quality evidence, benefits clearly outweigh risks and burdens or vice versa
Grade 2A	Weak recommendation, high-quality evidence, benefits closely balanced with risks and burdens
Grade 2B	Weak recommendation, moderate-quality evidence, benefits closely balanced with risks and burdens
Grade 2C	Weak recommendation, low-quality or very low-quality evidence, uncertainty in the estimates of benefits, risks and burden; benefits, risk and burden may be closely balanced

## Results

Two-hundred forty-seven articles were included in this review. Eighty-four recommendations were developed (Supplemental file 3).

## Discussion

### Injury patterns of patients with multiple trauma in the mountains

The injury patterns of multiple trauma vary according to the terrain (e.g. grassy or rocky ground), protective equipment and techniques (e.g. helmet, rope, belaying) and the activity. For instance, critical injuries in climbing often involve the pelvis and chest [7], canyoning accidents the lower and upper extremities [8], and mountain biking, winter, and aviation sport injuries the head and thoracolumbar vertebral column [9–11].

### Challenges in mountain rescue

Accidents in the mountains create challenges that are not found in most urban scenarios. Trauma victims can be difficult to locate and extricate because of the terrain. Bad weather can impede rescue efforts and limit the delivery of on-site patient care. Environmental factors may demand deviation from normal patterns of care provided in an urban environments. Evacuation to definitive care can be greatly delayed due to travel over difficult terrain and bad weather. Medical response in mountainous terrain can also be greatly delayed compared to an urban setting. Mountain victims may not receive care within the same time frame as in an urban setting because of the location. This article focuses on helicopter-supported mountain rescue missions because in many developed countries (e.g. Austria > 98%), as well as in some developing countries, the large majority of multiple trauma patients are rescued by HEMS. Ground-based mountain rescue services are still necessary during conditions that prevent helicopter flights, such as bad weather, darkness, when night vision goggles are not available, and high altitude. Technical possibilities and human resources may be very limited during ground rescue missions. In ground rescue missions, only basic equipment may be available. The physical and psychological challenges may be extraordinary. Trauma mortality is not necessarily higher with prehospital times longer than 60 min except for patients in haemorrhagic shock [12, 13]. The goal is to provide the best possible care throughout the rescue effort, given the unique situation and based on the principles described in these recommendations.

**Recommendations**: Consider terrain, weather, transport conditions and limited resources when treating a multiple trauma patient in the mountains (1C).

### Rescuer safety

**Fig. 1:**
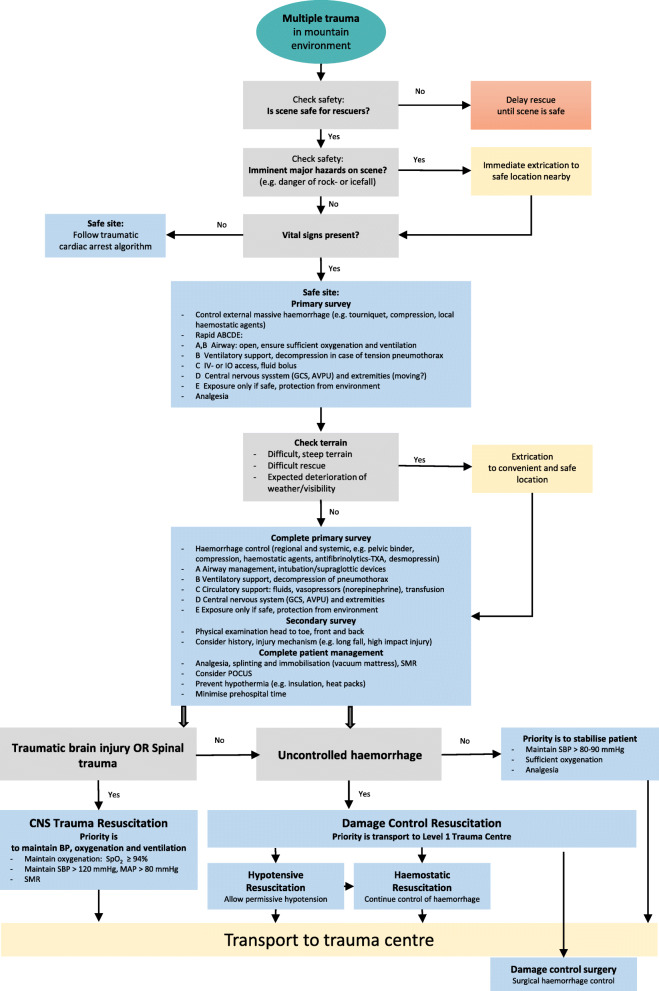
Treatment of multiple trauma in mountain environments algorithm. ABCDE; Airway, Breathing, Circulation, Disability, Exposure; IV; intravenous; IO; intraosseus; GCS: Glasgow Coma Scale; AVPU: Alert Voice Pain Unresponsive; TXA; tranexamic acid; SMR: spinal motion restriction; POCUS: point of care ultrasound; BP: blood pressure; SBP: systolic blood pressure; SpO_2_: oxygen saturation by pulse oximeter; MAP: mean arterial pressure; mmHg; millimetres of mercury

The safety of the rescuers is the first principle of rescue (Fig. 1). Rescuers should be able to move safely in hazardous mountain terrain. Helmets can reduce the likelihood and severity of traumatic brain injury (TBI). Mountain rescuers should wear helmets to protect against TBI and should use additional safety equipment to prevent injuries. On-site patient care may be hazardous to both patients and rescuers, because of steep or slippery terrain, rock-, ice- or snowfall, avalanches, and low visibility. Rescuers must consider potential hazards including transport to and from the scene, access to the scene, clothing and equipment for the rescuers, and reliable communications with other agencies and team members. Rescuers must also use personal protection from body fluid exposures and be prepared to handle immediate life threatening conditions. The decision to ‘stabilise on site and prepare for transport’ versus a ‘grab and go’ approach will necessarily be made on a case-by-case basis. In hazardous conditions, it may be necessary to evacuate a casualty from the accident site as rapidly as possible before any medical treatment has been given, even if there are critical injuries [14].

**Recommendations**: On-site safety of rescuers takes precedence over all other considerations (1C). Wear helmets to protect against TBI (1B) and use additional safety equipment to prevent injuries and infections (1C). In a hazardous environment, consider a strategy of ‘grab and go’ rather than ‘stabilise on site.’ (1C).

### Critical bleeding (Cr)

In contrast to patients with multiple trauma in military operations, patients in the mountains with multiple trauma very rarely sustain life threatening limb haemorrhage that can be treated with relatively simple measures, such as a tourniquet. Rather, these patients often sustain blunt injuries with bleeding from internal organ injuries that can only be treated surgically (e.g. by damage control surgery or interventional radiology).

In patients with multiple trauma, control of massive external haemorrhage with methods such as compression, haemostatic agents, or tourniquets, takes priority over other components of ABCDE because massive external haemorrhage leads to death most rapdily [15]. In tactical or military medicine this is known as the critical bleeding or the eXsanguination ABCDE approach [16] (crABCDE or XABCDE).

### Airway (A) and cervical spine (c-spine)

Maintaining an open airway is the second essential step in the treatment of trauma patients. Oxygen is recommended for trauma patients, especially at high altitude (above 2500 m), in order to preserve normoxia [17]. Available oxygen may be limited, especially during long or technically difficult rescues. A suction device must be ready at all times [18]. An obstructed airway requires immediate airway management. To open the airway, the initial action should be a jaw thrust, avoiding excessive movement of the cervical spine. Indications for advanced airway management include apnoea, agonal respirations, severe thoracic trauma, and TBI with seriously impaired gas exchange [18]. Advanced airway management prior to extrication by hoist or long line carries a risk of dislodging the airway device or hypoventilating the patient [19]. Providing a definitive airway can be difficult. Video laryngoscopy with a bougie may facilitate tracheal intubation [20]. Prehospital tracheal intubation should only be performed by experienced rescuers. In austere environments, use of supraglottic devices may be superior to tracheal intubation [21]. When a definitive airway is required and tracheal intubation is not possible, creation of a surgical airway with a cricothyrotomy may be necessary if supraglottic airway insertion and bag-valve mask ventilation fail. Ventilation with a bag-valve mask or a supraglottic airway is often as effective as tracheal intubation [22]. Immediately after establishment of an advanced airway (a tracheal tube or supraglottic airway), capnography should be used to confirm correct placement and to achieve normal ventilation. In prolonged rescue missions, bag-valve mask ventilation should only serve as a bridge to a protected airway.

Immobilisation of the c-spine is not necessarily recommended for all blunt trauma patients (Table 2) [24–26] and should not be performed in neurologically intact patients with penetrating trauma [23, 27–33]. A clinical decision rule, such as NEXUS or the Canadian C-spine Rule, should be used to avoid secondary spinal injury [33–36]. Prehospital clearance of the c-spine in children (≤8 years, although not uniformly defined) is not recommended [37–40]. Methods to immobilise the c-spine may include manual in-line stabilisation, SAM splints, or cervical collars [41]. Cervical collars must be applied correctly [42], with special attention to maintaining venous return [23, 43–47]. Spinal injury is covered under Disability.

**Table 2:** Patients who do not require immobilisation must meet all five of the following criteria [23]

• awake and alert,	
• not intoxicated	
• no painful distracting injuries	
• no tenderness at the posterior midline along the cervical spine	
• no focal neurologic deficit	

All other patients require spinal motion restriction with stabilisation of the entire body, unless they have an ABCD instability, in which case minimal spinal motion restriction and immediate transfer may be preferred

**Recommendations**: Provide oxygen (2B), especially at high altitude (above 2500 m) (1A). Rescuers should be competent in opening and clearing an airway, and in maintaining a patent airway (1C). Only experienced rescuers should perform prehospital tracheal intubation (1B). Consider advanced airway management if gas exchange is seriously impaired (2B). Be cautious with advanced airway management prior to extrication by hoist, because the artificial airway may be dislodged or the patient may be hypoventilated (1C). Consider using a video laryngoscope and an introducer to facilitate tracheal intubation (1B), or a supraglottic device as an alternative to tracheal intubation (2A) Tracheal intubation of a child should only be performed by an experienced rescuer. Otherwise, ventilate with a bag-valve mask (2B). After establishing an advanced airway, use capnography to confirm correct placement and to maintain normal ventilation (2B). Do not immobilise the c-spine of a blunt trauma patient who does not meet criteria for c-collar placement according to a validated decision rule (1A). Do not immobilise the c-spine of a neurologically intact patient with penetrating trauma (1A). Do not clear the c-spine in children in a prehospital environment (1C). The c-spine may be immobilised using manual in-line stabilisation, a SAM splint, or a cervical collar (1B).

### Breathing (B)

Normoxia and normocapnia are optimal to support physiological organ function. Ventilatory support is desirable if the patient is not able to maintain normoxia with supplementary oxygen or if hypercapnia may have deleterious effects, as in a hypoventilating patient with TBI. With the lowest possible oxygen flow to preserve supplies, aim for oxygen saturation ≥94% (88% in patients with chronic pulmonary disease). Avoid hyperoxia, as it may decrease survival [48]. Once an advanced airway is established, normal ventilation should be achieved with lung-protective ventilation according to ideal body weight, monitored by end-tidal carbon dioxide measurement (capnometry) and pulse oximetry [49]. Acute respiratory failure after severe trauma may be caused by a severe chest injury, such as flail chest, lung contusions or lacerations, or tension pneumothorax. Acute respiratory failure may also occur after TBI or spinal trauma, because of respiratory paralysis, aspiration, or airway obstruction secondary to decreased level of consciousness.

**Recommendation**: Establish normal ventilation with lung-protective ventilation and establish normoxia and normocapnia in TBI patients (1A).

### Thoracic injury

Multiple trauma is frequently associated with blunt thoracic trauma [50]. The main symptoms of blunt thoracic trauma are pain and difficulty breathing. Initial assessment should include pulse oximetry and assessment of breathing to identify respiratory distress [51]. Severe pain from fractured ribs may compromise ventilation. Effective analgesia and oxygen administration may improve ventilation and oxygenation. A noncritical pneumothorax or haemothorax may remain undiagnosed without risk to the patient. Consideration must be given to the expansion of trapped gas in the pneumothorax if the helicopter must gain substantial elevation during the evacuation [52]. If oxygenation does not improve or deteriorates and severe respiratory or circulatory compromise occurs, it is critical to diagnose a tension pneumothorax and to perform an immediate decompression of the pleural cavity [53]. Needle decompression in the second or third intercostal space in the midclavicular line can be rapidly and easily performed as the first step in treatment, but has a considerably higher failure rate than tube thoracostomy [54]. Tube thoracostomy is superior to needle decompression, although it is not without risks [53, 55]. Pigtail catheters are increasingly used because they are minimally invasive and have a lower complication rate than tube thoracostomy. They may become the prehospital intervention of choice in uncomplicated pneumothorax [56, 57]. The prehospital use of a minithoracostomy, a skin incision followed by blunt finger dissection without a trocar, may have the lowest complication rate, but can create a sucking chest wound in a nonventilated patient [58]. Massive haemothorax may cause both severe respiratory distress and significant blood loss, necessitating immediate evacuation to a trauma centre. In several HEMS systems, thoracostomies are performed routinely in anaesthetized patients receiving positive pressure ventilation. In remote mountain emergency operations, during a long rescue, a thoracostomy may be required as a lifesaving procedure without positive pressure ventilation. If a thoracostomy is performed on a patient without a tension pneumothorax, negative pressure ventilation (spontaneous breathing) may lead to accumulation of a simple pneumothorax with respiratory compromise.

**Recommendations**: Identify respiratory distress and use a pulse oximeter (1B). Consider the potential critical expansion of a pneumothorax during helicopter evacuation when substantial elevation gain is necessary (1B). If severe respiratory or circulatory compromise occurs, consider the cause to be a tension pneumothorax. Immediately decompress the pleural cavity (1B) with a minithoracostomy (1B) or pigtail catheter (2B).

### Circulation (C)

Severe haemorrhage is the second leading cause of death, after TBI [59]. Decreased cardiac output and blood pressure reduce tissue oxygen delivery.

#### Therapeutic targets

Prioritise haemorrhage control. Maintain oxygenation and perfusion using clinical and ultrasonographic findings [60] with a goal of mean arterial blood pressure (MAP) ~ 65 mmHg in previously normotensive patients [61, 62].

#### Monitoring in mountain environments

Pulse oximetry and blood pressure often are readily obtainable. Perfusion can be measured clinically by assessing consciousness and capillary refill (limited in cold and with anaemia). Blood pressure measurement only provides a surrogate marker of perfusion and oxygen delivery. With noncompressible haemorrhage, allow for permissive hypotension [61] and prioritise rapid transport.

### Bleeding control

#### Nonpharmacologic methods

First, attempt direct manual compression. Continued extremity bleeding should be controlled with a tourniquet [63–65]. Modern tourniquets can decrease haemorrhage, prevent shock, decrease limb loss caused by ischaemia, and permit rapid extrication [66, 67]. Mortality increases if tourniquet use is delayed until trauma-centre arrival [68]. Windlass-style tourniquets (eg CAT or SOFTT tourniquets) are optimal. Tourniquets are superior to direct pressure in severe extremity exsanguination. Usually they are left in place for 2-6 h [61]. It is best to release the tourniquet only after arrival to definitive care. Tourniquet-related complications are rare for tourniquets applied less than 2 h. Complications may also be related to degree of tissue injury [69]. There are anecdotal reports describing complications. Some guidelines recommend checking for bleeding every two hours [70]. Control of noncompressible haemorrhage with expandable sponges is a novel method [71]. Junctional tourniquets may control haemorrhage in inguinal and axillary areas where standard tourniquets are not effective [72]. Temporary aortic occlusion with resuscitative balloon occlusion of the aorta (REBOA) has been described for internal abdominopelvic haemorrhage, but substantial training and resources are required [73]. REBOA is a complex technique, which only a few highly advanced HEMS may be able to offer. The use of ultrasound may make REBOA more accurate and safer. The technique and pitfalls have been reviewed in detail elsewhere [74]. Pelvic binders close the pelvic ring [75, 76]. Pelvic binders may have an effectiveness of 70% in stabilizing the pelvis [77, 78]. Training is required. Binders must be carefully positioned to be effective.

#### Pharmacologic methods

##### Antifibrinolytics

Coagulopathy increases mortality in severe exsanguination. Haemorrhagic death [79–84] and multi-organ failure with severe shock [85] decrease with administration of tranexamic acid TXA) within 3 h after trauma, without increasing the risk of thrombosis [79]. The dose is 1 g intravenously (IV) over 10 min, followed by 1 g IVover 8 h [61].

##### Platelet aggregators

Desmopressin enhances platelet aggregation. It is helpful for patients on platelet inhibitors [86–93] or with von Willebrand disease [94]. The dose is 0.3 mcg/kg IV over 30 min [95]. Desmopressin should not be given routinely in bleeding trauma patients, but should be considered in patients with hypothermia-induced coagulopathy [96]. Desmopressin increases platelet aggregation in acidotic hypothermia [97]. In isolated hypothermia, the recommended dose is 1.5 mcg subcutaneously [96]. With acidosis and hypothermia the dose is 0.3 mcg/kg IV [97]. Desmopressin does not increase the risk of thrombotic events [98].

##### Factor concentrates

Reversal of trauma-induced coagulopathy with fresh frozen plasma (FFP) can fail from lack of fibrinogen. Empiric administration of 3 g of fibrinogen concentrate IV decreased mortality in multiple trauma in a Japanese study [99]. Fibrinogen concentrate may decrease blood transfusion needs, multi-organ failure and mortality [100]. Reversal of vitamin K inhibition with four-factor prothrombin complex concentrate, at a dose of 25 U/kg IV, is promising for geriatric trauma patients, with or without intracranial haemorrhage, on warfarin or direct oral anticoagulant therapy [101–104].

Haemostatic dressings are superior to plain gauze. Biocompatible chitosan-impregnated gauze (Celox or ChitoGauze Pro) is best. It links platelets and red blood cells, forming a mucoadhesive barrier after 2-5 min of direct pressure [105]. HemCon bandages are less effective in complex wounds. Zeolite granules are rarely used because of possible exothermic reactions. Zeolite sponges have a lower risk of burns and are less likely to embed inorganic material in the wound [106] (Table 3). Recently, bioabsorbable cellulose (e.g. WoundClot), with embedded coagulation factors, has been introduced. The haemostatic effects are promising [107].

**Table 3:** Summary of haemostatic bandages, adapted from [105]. Gen denotes generation, RBCs red blood cells

Manfacturer	Gen	Mechanism of action	Form	Application
Celox gauze, MedTrade Products Ltd., Crew, UK	3rd	Cross-links RBCs to form mucoadhesive barrier	Chitosan rolled gauze Z-fold, 3 in. × 10 ft	Packed into wound, 3 min direct pressure
CeloxGauze Pro, HemCon Medical Technologies, Portland, OR	3rd	Cross-links RBCs to form mucoadhesive barrier	Chitosan gauze Z-fold, 12 ft. length	Packed into wound, 2-5 min direct pressure
XStat, RevMedx Inc., Wilsonville, OR	3rd	Cellulose sponges coated with chitosan to assist with a mucoadhesive barrier	92 flat, circular, compressed mini sponges packaged in a 60 mL syringe applicator	The applicator has a small diameter insertion device available for use in wounds with narrow wound tracts

### IV access

At least one large bore IV is required in a multiple trauma patient who will require large volumes of fluid. Intraosseous volume replacement is slower. When fluid and drug administration are necessary, IO access should be obtained if IV access cannot be established after three attempts.

### Volume replacement, blood products, and vasopressors

Rapid volume replacement (Table 4) can restore cardiac preload and mitigate the effects of haemorrhage, if provided judiciously. Helicopters have transported uncrossmatched O negative packed red blood cells (PRBCs) to mountain rescue and other prehospital scenes. No transfusion reactions have been reported. Improved outcomes have not been reported in civilian rescue [108–111], but are common in military medicine [112]. In a TBI patient without critical bleeding, vasopressors may help maintain cerebral perfusion pressure if MAP decreases during prolonged transport. Norepinephrine is the preferred vasopressor. It should be given via a large peripheral vein, Uses of fluids and other adjuncts for haemorrhagic shock are shown in Table 4 [61, 66, 113].

**Table 4:** Fluid resuscitation and adjuncts in haemorrhagic shock. AKI: acute kidney injury; CNS: central nervous system; HES: hydroxyethyl starch solution; ICU: intensive care unit; MA: metabolic acidosis; PRBCs: packed red blood cells; TBI: traumatic brain injury

Agent	Advantages	Disadvantages	Notes
**CRYSTALLOIDS** 0.9% Normal Saline (‘unbalanced’)	Readily available, familiar; compatible with most medications and blood products	Not ‘physiologic’ (high chloride load); excess administration leads to AKI and MA (2C)	Bolus to effect after bleeding controlled. (1A)
Ringer’s lactate/ acetate (‘balanced’); Plasmalyte	Readily available; ‘physiologic’	Slightly hypotonic; excess administration worsens TBI (1C)	May reduce incidence of AKI and mortality in ICU. Bolus with control of bleed (1A)
Hypertonic saline solution	Low weight and volume (easier to transport); thermal stability; safe	May interfere with coagulation in patients with severe TBI	NaCl concentration > 0.9%; may expand volume, no long term survival benefit or improved CNS outcome vs. NaCl 0.9%
**COLLOIDS** Albumin, hydroxyethyl starch (HES), Dextran	Used as volume expander	Expensive; no proven mortality benefit. HES may increase harm in some subgroups.	Prehospital data still rare. HES may impair coagulation
**PACKED RED BLOOD CELLS / PLASMA / WHOLE BLOOD**	May improve survival or physiology, for Hb < 7 g/dL; lyophilised plasma is used in damage control	Inconvenient in out-of-hospital environment; ARDS/ transfusion reactions	Used by few centres; PRBCs:plasma: platelets 1:1:1 or 2:1:1 (1B), or fibrinogen 0.5 g per unit PRBCs (1C) in hospital
**VASOPRESSORS**	Use after adequate volume replacement (1C), Push-dose pressors simple; cardiac dysfunction: epinephrine	Does not treat cause; uncertain long-term benefit; dosing errors,; uncertain benefit (haemorrhage)	Constricts capacitance vessels; used in airway management / TBI with hypotension

#### Recommendations

**General principles**. Stop haemorrhage (1A) and maintain oxygenation and perfusion (MAP ≥65 mmHg in previously normotensive patients) (1C). With uncontrolled haemorrhage, allow permissive hypotension (1B). Rapid transport may be critical (1B).

**Bleeding Control. Nonpharmacologic methods.** First, attempt direct manual compression (1A). For uncontrolled extremity bleeding use a modern tourniquet with a windlass to control bleeding (1B) and facilitate extrication (1B). Release the tourniquet only after arrival to definitive care (2B). Do not release the tourniquet to check bleeding. (2C). Consider control of noncompressible truncal haemorrhage with expandable sponges and junctional tourniquets for axillary and inguinal areas (2C). For pelvic fractures, use a pelvic binder to close the pelvic ring (2C).

**Bleeding Control. Pharmacologic methods.** Administer TXA within 3 h post-trauma (1B). Consider desmopressin for patients on platelet inhibitors, with von Willebrand disease (2A), and with hypothermia-induced coagulopathy (2C). Consider fibrinogen concentrate administration rather than fresh frozen plasma (FFP) (2B). Consider prothrombin factor concentrate administration (2A). Consider reversal of vitamin K inhibition (2A) or direct oral anticoagulant therapy (2C). Consider using haemostatic dressings rather than plain gauze (1C).

**IV access, volume replacement, and vasopressors**. Establish at least one large bore IV for administration of fluid (1C). Establish IO access if IV access is impossible after three attempts when fluid and drug administration are required (1C). Use judicious rapid volume replacement to restore cardiac preload (1A), but be aware of detrimental effects of cold fluids such as dilution and hypothermia-induced coagulopathy. Consider norepinephrine in TBI patients without critical bleeding to maintain cerebral perfusion pressure during prolonged transport (1C).

### Disability (D)

#### Traumatic brain injury

The combination of TBI and multiple trauma is a predictor of poor outcome [114].

#### Airway management

Increases in the severity and duration of secondary insults correlate with worse outcomes [115]. Maintaining an open airway may help to minimise secondary brain injury. In severe TBI, supplementary oxygen may help to avoid secondary brain injury. If the rescuers have only basic skills, simple airway procedures may improve survival [116]. Tracheal intubation has caused increased mortality in some trauma systems, probably because rescuers lacked adequate airway management skills [116–119]. Improved outcomes have been reported for tracheal intubation in severe TBI when experienced providers deliver care using rapid sequence intubation with neuromuscular blocking agents [119–121].

#### Ventilation and oxygenation

Ventilation should be assessed clinically (rate, depth, effort). Patients should be monitored by pulse oximetry and waveform capnography. Hypoxia (SpO_2_ ≤ 94%), hyperventilation (ETCO_2_ < 35 mmHg - < 4.5 kPa) and hypoventilation (ETCO_2_ > 45 mmHg - > 6 kPa) are associated with worse outcomes [122, 123]. Achieving normocapnia in patients with multiple trauma can be challenging. Continuous monitoring with capnography can reduce hypo- and hyperventilation in TBI patients [49, 115, 124]. Rescuers should attempt to maintain normoxia (SpO_2_ 95-98%) and normocapnia (ETCO_2_ 35-45 mmHg - 4.5-6 kPa at sea level).

#### Hypotension

It is essential to control haemorrhage in order to minimise secondary TBI caused by hypotension. The injured brain loses autoregulation, resulting in secondary ischaemic damage. Hypotension (systolic blood pressure [SBP] < 110 mmHg) increases morbidity and mortality in TBI [125]. Mortality is higher when hypoxia and hypotension are combined [115]. Outcomes worsen with more episodes, increased severity, and longer duration of hypotension. In TBI, the traditional definition of shock (SBP < 90 mmHg) underestimates the effect of hypotension [126–128]. Patients with moderate to severe TBI should be considered hypotensive with SBP < 110 mmHg [127, 128]. In a retrospective registry study, mortality increased 4.8% for every 10 mmHg decrease in SBP when SBP was < 110 mmHg [129, 130]. In critical patients with TBI, a target SBP of 120 mmHg effectively minimised secondary insults [131]. The target SBP for cerebral resuscitation in TBI should be ≥110 mmHg. In multiple-trauma patients with TBI, the need to maintain cerebral perfusion pressure with increased SBP conflicts with the use of permissive hypotension. This conflict has not been studied in adults. In children timely haemodynamic resuscitation, to treat TBI, improves outcomes [132]. The use of hypertonic saline rather than standard fluids does not improve outcomes [133]. In the mountains, especially on ground rescue missions, small volumes of hypertonic saline may be more practical than standard fluids [134]. The value of prehospital vasopressors remains uncertain [135], but they may be helpful to maintain adequate SBP. Temperature management to avoid hypothermia improves haemorrhage control to minimise secondary injuries [136].

#### Methods to decrease intracranial pressure (ICP)

Use of mannitol or hypertonic saline can reduce ICP temporarily, for similar periods of time [136–138] Neither improves outcomes [139]. Elevation of the head to 30° may decrease intracranial pressure in paediatric TBI [140], A Cochrane database systematic review concluded that the evidence to support beneficial clinical outcomes was very low quality [141]. Head elevation is the preferred position, but should not delay timely rescue and evacuation [141].

#### Tranexamic acid to decrease haematoma expansion

The use of TXA within 3 h of TBI decreases mortality without increasing adverse events [142, 143].

#### Hypothermia and TBI

Hypothermia is independently related to poor outcomes in TBI [144, 145]. Increased mortality with hypothermia in multiple trauma is likely related to coagulopathy causing increased haemorrhage and hypotension, with decreased cerebral perfusion pressure. There is no evidence to support the use of early therapeutic hypothermia in the management of TBI [146–148].

**Recommendations**: Assess ventilation clinically (1C) and monitor patients with pulse oximetry to minimise hypoxia (1C). Use capnography to maintain normocapnia (1C). Maintain systolic blood pressure ≥ 110 mmHg (2C). Expedite rescue. Do not delay evacuation by attempting to maintain elevation of the head (1C). Administer TXA within 3 h after trauma (1A). Avoid hypothermia (1C).

### Spinal injury

Careful positioning and transport of patients with spinal injury in rough terrain may be challenging, but is important to avoid secondary injury. Spinal injury can involve only the bony spinal column or can be associated with spinal cord injury. Secondary insults result from movement, hypoxia, hypotension, and haematoma compressing the spinal cord. There are questions about the efficacy and safety of traditional spinal immobilisation for all trauma patients [149–152]. A more selective approach may be better [149, 153, 154]. Spinal motion restriction in the mountains may interfere with life saving interventions and may delay transport to definitive care. Spinal motion restriction is necessary for any patient with an altered level of consciousness [155]. A clinical decision rule should be used to identify patients at risk of significant spinal injury [33–36].

Manual in-line stabilisation with a ‘trapezius squeeze’ hold is as effective as a cervical collar to protect the c-spine during movement and during tracheal intubation [156]. Cervical collars are not necessary for all patients. They do not eliminate all movement. Adverse effects, including interference with breathing and venous compression may contribute to physiologic compromise of a patient with multiple trauma [25, 44, 157, 158]. Spinal motion restriction can be achieved using a combination of manual in-line stabilisation, head blocks, and hard or soft transfer devices [159]. Unstable patients with multiple trauma should be handled as little as possible. Logrolling is of limited diagnostic value in most circumstances. Use of a manual vertical lift or a scoop stretcher limits spinal motion [160]. A vacuum mattress is a stable transfer device that provides comfort and protects against pressure necrosis during prolonged transport [161–164]. For extrication and transport, a vacuum mattress should be used in a horizontal rescue bag or stretcher. When horizontal extrication is not possible, as in narrow crevasses, a Kendrick Extrication Device (KED) or newer devices such as NEXT can be used to stabilise the spine in a sitting position [165].

**Recommendations**: Immobilise the spine of all multiple trauma patients with altered level of consciousness (1C). Use a clinical decision rule to identify patients at risk from secondary spinal injury and only if positive immobilise the spine (1B). Limit spinal motion with a combination of manual stabilisation, head blocks, and hard or soft transfer devices (1C). Choose techniques that require minimal handling (1C). Do not log roll unstable trauma patients (1C). Consider a vacuum mattress for a long comfortable transfer (1C). Consider a KED or similar device for extrication in a non-horizontal position (1C).

### Environment and exposure (E)

Prehospital assessment of the type and severity of multiple trauma should guide treatment, including the choice of destination, especially in life-threatening cases. Physical examination may be limited by adverse environmental conditions [166] and cannot be performed reliably without some degree of exposure. Exposure may cause or worsen hypothermia, a common condition in trauma patients that contributes to coagulopathy and acidosis [166–168]. History and physical exam alone have a low accuracy in detecting injuries in blunt trauma, missing almost half of injuries, even in a hospital environment [169]. Prehospital injury assessment by physicians can miss up to one-third of significant injuries [170]. The value of exposure should be weighed against the risk of hypothermia. Examination should be performed sequentially, by body region, avoiding heat loss and preserving insulating clothing.

**Recommendations**: Consider whether exposure will be helpful. (1C). Sequentially examine by body region, avoiding heat loss and preserving insulating clothing (2C).

### First aid, splinting and immobilisation

Injuries of the extremities are the most common cause of evacuation by organised mountain rescue services in Europe and the US. In a survey from Scotland, 50% of the survivors suffered from lower limb trauma [2]. Similarly, in the US, sprains, strains, and fractures were the most common medical incidents amongst recreational wilderness medicine expeditions, with fractures being the most frequent cause for evacuation [171]. In a multiple trauma patient, extremity injuries may be associated with additional life-threatening injuries. Lifesaving interventions, folllowing crABCDE, should precede other care. Extremity injuries that are not life threatening, should be immobilised only after the patient has been stabilised [14].

Although splinting and immobilisation have been described since ancient times, accepted practise has been established more by time than by high quality randomised controlled trials. Benefits of fracture reduction and immobilisation include pain control, decreased blood loss, prevention of conversion from closed to open fracture, and protection from further injury [172]. Early immobilisation, reduction, and splinting reduce pain in patients with closed fractures treated in the emergency department. Splinting can cause harm if not done correctly. Some interventions, including traction splints for femur fractures, may cause morbidity even when properly applied. In patients with multiple trauma, contraindications to traction splinting for femur fractures are common. Contraindications include non-midshaft location and associated knee or tibia-fibula fractures. Most patients with multiple trauma should be extricated on vacuum mattresses or spine boards [173, 174]. Spine boards have hard surfaces and should only be used for extrication. They are not suitable for transport. Spine boards can cause tissue necrosis even when used for relatively short time periods. Patients with hypotension or hypothermia may be at increased risk of tissue necrosis.

Immobilisation of extremity injuries should follow standard splinting procedures, including the use of sufficient padding, immobilising the joints above and below the injury, and neurovascular checks before and after splinting. Traction splinting for midshaft femur fractures should be used only if needed for pain control, haemorrhage reduction, or for immobilisation if simple splinting does not suffice. The choice of splinting device is subject to many factors including the part of the body to be splinted, cost, weight, compatibility with other rescue equipment, and regional preferences (Table 5) [175]. Whilst there have been no randomised comparisons of splinting devices,vacuum splints have been proven effective and, despite their weight, have been widely adopted [163, 165, 176, 177].

**Table 5:** Benefits of reducing and immobilising a fracture, adapted from [14]

Reduced pain	
Reduced blood loss	
Minimised neurovascular complications	
Reduced risk of fat embolism	
Reduced risk of further tissue damage; facilitated healing	
Easier transport	

**Recommendations**: Consider early splinting to reduce pain and blood loss and to facilitate transport (1C). Use splinting devices with which you are familiar (1C). Consider the use of vacuum splints (1C). Transport patients with multiple trauma using vacuum mattresses, rather than spine boards, to avoid additional soft tissue injury (1C).

### Analgesia

Multiple trauma patients in the mountains should receive adequate analgesia. Analgesia decreases acute and long-term physiological and psychological responses to the stress of trauma [178, 179], increases comfort, and facilitates evacuation. Depending on training and licensure, mountain rescuers should be well versed in various modalities for pain reduction [178, 180]. Approaches to analgesia for trauma patients in the mountains vary widely amongst countries [178]. This is the result of differing professions and skill levels of medical providers, diverse laws and regulations, and large variations in transport times, especially with HEMS as opposed to ground rescue.

**Nonpharmacologic interventions** for acute pain in the mountains may include distraction and hypnosis. Most evidence is based on case reports. A recent meta-analysis demonstrated that distraction techniques, especially virtual reality and hypnosis, were moderately effective for pain relief in adults undergoing procedures for burn wound care [181]. Minor pain from trauma, is usually amenable to conservative treatment. Guidelines have been published by the Wilderness Medical Society for ‘PRICE’ therapy [180].

- Protect the injury (immobilise)
- Rest
- Ice (attenuates inflammation)
- Compression bandage
- Elevate the extremity

#### Systemic analgesia

Because opioids may worsen haemodynamics and depress ventilation, they should be used carefully. Judicious use of opioids may prevent untoward effects. Adverse effects can be reversed by naloxone. The use of ketamine and other medications, alone or with opioids, can prevent haemodynamic and respiratory decompensation (Table 6).

**Table 6:** Systemic analgesics, adapted from [180]. LOE denotes level of evidence in parentheses, provided if available. COX cyclooxygenase, mcg micrograms, mg milligrams, g grams, kg kilograms, mL milliliters, IV intravenous, IM intramuscular IN intranasal, NSAIDS nonsteroidal anti-inflammatory drugs, OTFC: oral transmucosal fentanyl citrate, po: by mouth, q: ‘every,’ qd: daily, bid: twice daily, tid: three times daily

AGENT/DOSE SITE	DOSAGE ADULTS/(PEDS); LOE	REMARKS
**Ice**	1B	Simple, noninvasive; reduces inflammation/oedema; avoid freezing injury [180].
**NSAIDS/paracetamol**	1A	All NSAIDS: if po, potential dyspepsia lessened with food. Avoid with GI bleed/ulcer history, dehydration. Possible kidney injury or increased bleeding
Diclofenac topical	2.3% topical; 2-4 g bid; unknown	
Ibuprofen PO	2400 mg/d divided tid (10 mg/kg/d); 1A	
Naproxen PO	660 mg/d divided tid; unknown	
Meloxicam PO	15 mg qd; unknown	Cardiovascular events may increase. COX-2 selective inhibitor meloxicam minimises bleed/platelet disfunction.
Ketorolac IM	60 mg q 6 h (0.5 mg/kg q6h); 2C	
IV	15-30 mg (0.5 mg/kg, max 15 mg); 1B	
Paracetamol PO	Max 1300 mg (10 mg/kg) TID; 1B	Renal and GI sparing. Avoid in severe hepatic disease. Overdose can result in hepatic failure [179, 180, 182].. NSAIDS + paracetamol result in pain diminution better than either alone.
IV	> 50 kg:1 g < 50 kg:15 mg/kg IV/15 min; 1B	
**OPIOIDS**		All opioids tend to cause respiratory depression/desaturation and arterial hypotension; monitor. Avoid opioids if patient needs full cognition (i.e. self-evacuation). Naloxone reverses opioids [178–180].
Fentanyl IV	25-100 mcg (1-3 mcg/kg); 1A	Slow fentanyl push mitigates risk of ‘frozen chest.’
IN	180 mcg (1.5 mcg/kg); 1B	
Buccal/transmucosal	OTFC: 800 mcg (10-15 mcg/kg); 1B	Oral transmucosal fentanyl citrate self-administered, ideal for austere situation. Transdermal route good for sustained dosing.
Transdermal		Transdermal route good for sustained dosing.
Morphine IV	5-10 mg (0.1 mg/kg-max 10 mg); 1A	Avoid morphine in renal failure. May cause histamine release.
IM	10-20 mg (0.2 mg/kg, ma× 10 mg); 2B	Poor blood flow may limit absorption.
Oxycodone PO	5-10 mg q8 h; 2B	PO opioids easy to carry on smaller expeditions.
**OTHER**		
Ketamine	1B	Use half dose for S-ketamine. Slower administration lessens emesis and psychosis. Can cause hypertension and tachycardia; preserves respiration; many prefer for multiple trauma. Vocal calming measures and adding midazolam minimise psychosis [178–185].
IV	10-30 mg (0.1-0.3 mg/kg); 1B	
IM	1 mg/kg; 2C	
IN	0.5 mg/kg (0.5 mg/kg); 2B	
Methoxyflurane Inhaled	3 mL given to self; max 6 mL/day; 2A	Altitude use. No renal effects; avoided by some; anxiolysis [186–188].
Nitrous Oxide nhaled	60-70% N_2_O/40-30% O_2_; 2B	Less effective at altitude, complex; potentiates barotrauma!

#### Regional Anaesthesia

Regional anaesthesia can be used in a mountain environment, especially to treat painful injuries during prolonged, difficult extrications (Table 7). Regional anaesthesia can decrease the need for systemic analgesia [195]. Regional anaesthesia involves special techniques. A practitioner must be fully aware of the indications, contraindications, and possible complications and should discuss the risks and possible benefits with the patient, if possible, before proceeding. Regional anaesthesia can prevent respiratory or haemodynamic compromise that commonly occurs with the use of systemic analgesia [196]. The amount of local anaesthetic may need to be decreased in elderly patients and in patients with liver or kidney disease [197]. Ultrasound guidance increases the success rate and can help to limit the dose of local anaesthetic.

**Table 7:** Regional blocks appropriate for wilderness are listed below, adapted from [180], LOE denotes level of evidence

Regional block	Indications	LOE	Remarks
Intra-articular injection	Shoulder dislocation	2B	Not superior to procedural sedation [189]
Intrascalene nerve block	Shoulder/arm injuries	1C	Phrenic nerve paralysis and respiratory compromise (not ideal for altitude) [190]
Supra- or infraclavicular block	Pathology distal to shoulder	1B	Small ultrasound probe; 20-25 mL local anaesthetic, pneumothorax
Axillary block	Pathology distal to shoulder	1C	Less anesthetic needed with ultrasound (~ 15 mL) [191]
Median/ulnar/radial block	Distal forearm/hand/multiple	1C	Nerves of mid-forearm readily seen with ultrasound; 3-5 mL [190]
Intercostal nerve block	Isolated rib fracture(s)	1C	Ideal if cardiorespiratory status with systemic analgesia worrisome [192]
Femoral nerve block	Femur fracture/pathology	1C	Not effective for posterior limb, or distal leg
3-in 1 block	Femur/knee or distal extremity	1C	For thigh and distal extremity/foot; lateral femoral cutaneous- femoral and obturator nerve [191, 193]
Fascia Iliaca block	Femur fracture/pathology	1C	90% success rate prehospital, simple; less injury risk to nerves; ~ 30 mL [194]
Sciatic nerve block	Posterior thigh/knee/distal lower extremity	1C	With femoral and saphenous nerve block, good for knee and distal lesions [191]
Ankle nerve block	Ankle/foot	2B	Need to block 5 nerves; high failure rate, good for isolated foot lacerations

**Recommendations**: Consider **nonpharmacologic interventions (1C).**

#### Systemic analgesia

Consider ketamine and other nonopioid analgesics (1C). Use opioids judiciously (1C).

#### Regional anaesthesia

Consider regional anaesthesia in the mountains, especially for painful injuries during prolonged, difficult extrications or during fracture and joint reduction (1B). Regional anaesthesia may be indicated to avoid haemodynamic or respiratory compromise associated with systemic analgesia (1B). Ultrasound guidance is recommended (1C).

### Hypothermia and temperature management

Hypothermia is often associated with trauma [198, 199] and may increase mortality. For trauma patients, prevention of heat loss and rewarming can be critical. Prehospital recognition of hypothermia can be challenging. Clinical signs of hypothermia may be unreliable when there are coexisting conditions and when minimally invasive temperature measurement is not possible [200]. If a suitable thermometer is not available, core temperature can be estimated using the Swiss staging system [201]. The ideal prehospital thermometer should be easy to handle, accurate in all environmental conditions, and able to reflect small temperature changes rapidly. A good example is a thermistor-based tympanic probe designed for field use in cold environments (Table 8) [200]. The most accurate method of core temperature measurement is an oesophageal probe in the lower third of the oesophagus. This technique is only advisable when the airway has been secured.

**Table 8:** Different sites for temperature measurement, advantages and disadvantages for field use [200]

	Advantages	Disadvantages	Suitability for prehospital use	Logistic considerations	Field tested
**Skin (heat flux)**	Noninvasive	Low correlation with core temperature	High	Skin temperature affected by environment, e.g. cold or wet	Yes (experimental animal model)
**Epitympanic**	Minimally invasive. Correlates with brain temperature	Influenced by ambient temperature and insulation of ear canal. Affected if ear canal contains water or snow [202].	Moderate-high	Insulation of the external auditory canal improves the reliability of the reading. Thermistor technology ideal; infrared technology not reliable	Yes
**Rectum**	Commonly used in hospital	Lags behind core temperature when rewarming	Moderate	Needs to be inserted deeply (> 15 cm) to avoid measuring temperature of cold feces	Yes
**Bladder**	Allows to monitor urinary output	Can be affected by cold diuresis. Impractical for field use	Low	Mostly monitor based probes	No
**Oesophageal**	Best correlation with core temperature	Requires an advanced airway in place. Needs to be positioned in lower third of oesophagus for reliability	Moderate	Mostly monitor based (only one hand-held device)	Yes

A systematic review that assessed different types of insulation and active warming methods for use in a ‘hypothermia wrap’ concluded that there is a lack of prehospital randomised controlled trials with large sample sizes to provide strong recommendations regarding the most effective treatment in hypothermia [203]. It is not clear whether wet clothes should be removed before applying a tightly fitting vapour barrier [204, 205]. Wilderness Medical Society guidelines suggest cutting off wet clothing when a vapour barrier is not available or when the patient is at high risk of continued cooling [206]. The use of a vapour barrier, non-breathable waterproof material, to reduce evapourative and convective heat loss, is most effective when the vapour barrier, made of thick material or containing trapped air, (e.g. bubble wrap), is combined with thick insulating material [207–209].

Sources of external heat include heat packs (electrical or chemical), hot water bottles, heat blankets (chemical or electrical), and forced warm air. External heat should be used to prevent heat loss and for rewarming when endogenous heat production is decreased, because shivering is impaired or absent. Shivering is suppressed or abolished in patients with moderate or severe hypothermia, and in patients multiple trauma, even if they are not hypothermic. The use of external heat sources in mildly hypothermic patients without trauma reduces peripheral cold stress and shivering [209]. External heat also decreases the cardiovascular and respiratory stress of shivering and increases thermal comfort. Overall, external heat is recommended. Heat transfer is most efficient when external heat is applied to the axillae, chest, and back [210]. Exothermic chemical heat packs should be used cautiously if combined with supplemental oxygen. Higher oxygen levels can intensify the heat production, potentially causing thermal burns [211]. Intravenous fluids should be warmed to 40 °C. Portable battery-powered intravenous fluid warmers are the most practical heating devices. Caution is warranted in selecting the device. Most devices currently on the market are incapable of heating adequate amounts of cold fluids to 40 °C [212]. A temporary, warm microenvironment can be created by using a lightweight, rapidly deployed shelter. to decrease heat loss. Use of a warm environment also increases fine motor coordination for the rescuers.

**Recommendations**: If a suitable thermometer is not available, use the Swiss staging system to estimate core temperature (1C). Epitympanic thermometers using a thermistor are reliable for core temperature monitoring if they are designed for field use in cold environments. There is currently limited availability of suitable thermometers. (1B). In a patient with a secured airway consider core temperature measurement using an oesophageal probe (2A). A hypothermic patient should be extricated from the cold environment and covered by a vapour barrier and whole-body insulation (1C). Rewarming devices should be used in conjunction with vapour barriers and insulation (1C).

### Transport and hospital selection

A patient with multiple trauma should be transported as rapidly as possible to a high-level trauma centre. Transport decisions for a patient with multiple trauma in the mountains should be coordinated with assertive management in the field and should be made early in order to ensure rapid, definitive in-hospital care. A helicopter should transport a medically qualified team and equipment to the scene [213]. During transport, continuous monitoring and uninterrupted treatment should be continued. A lightweight, stable stretcher should be used. The patient should not be moved in a vertical position, because this may cause hypotension especially in a patient with significant haemorrhage. Helicopters provide gentler and more stable transport than ground ambulances. Gentle transport is especially advantageous for a patient with a spinal injury or haemodynamic instability [214, 215]. Shorter transport times improve outcomes for trauma patients in shock. Air transport may improve survival compared with ground rescue in patients with TBI [216]. Patients with multiple trauma and TBI should be transported by air when this minimises the time to definitive care. With short distances, helicopter transport may not save time compared to ground ambulance transport and may not improve outcomes [217–219]. Helicopter transport may minimise prehospital time and the length of exposure to austere conditions compared to ground transport. However, in the mountains, availability of helicopter rescue may be limited by weather, low visibility, high altitude, and sometimes by other technical considerations. The potential benefits of air rescue should be carefully weighed against the risks. Helicopters may provide opportunities to reach distant trauma centres within a reasonable time, bypassing local smaller hospitals [220, 221]. In densely populated, highly developed areas, such as the European Alps, most locations are within a reasonable distance from a high-level trauma centre (i.e. 15-20 min flight time). This is usually not the case in less densely populated and less developed areas. The benefits of air rescue should be balanced against the inherent risks, especially when flight conditions may be hazardous.

**Recommendations**: Expedite transport (1C). Use a lightweight and stable stretcher(1C). Avoid moving a patient in a vertical position, as this may cause hypotension, especially with significant haemorrhage (1C). Use helicopter transport for a patient with spinal injury or haemodynamic instability (1C). Transport TBI patients by air if this saves time to definitive treatment (1B). Use a helicopter to reach a distant high-level trauma centre, bypassing smaller local hospitals (1B). Balance the benefits of air rescue with the inherent risks (1C).

### Ultrasound

**Fig. 2:**
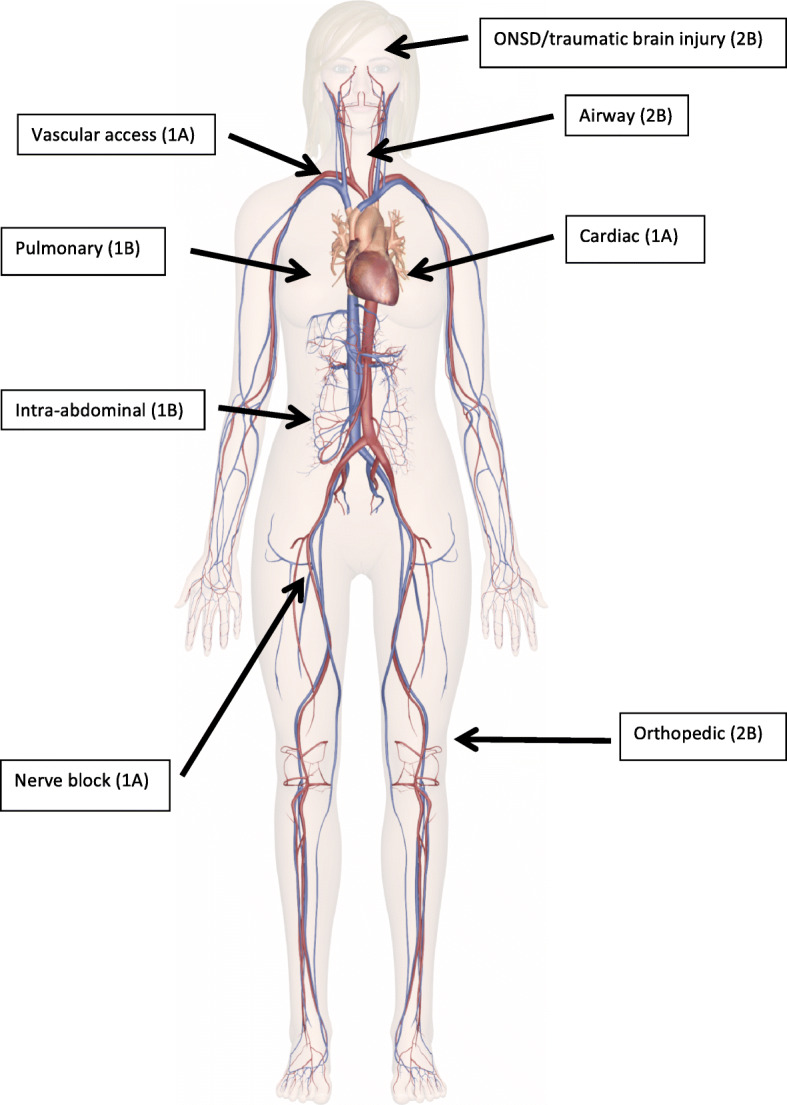
Use of ultrasound in multiple trauma patients and grading of evidence. ONSD denotes optic nerve sheath diameter

Point of care ultrasound (POCUS) is a key method for the initial assessment of patients with suspected trauma in the emergency department (Fig. 2) [222]. Trauma ultrasound (US) can be performed on helicopters [223] and in ambulances [224]. Ultrasound can be useful in trauma patients [225, 226], because it may help to guide treatment and the choice of destination hospital [227]. An operator should consider whether the results of POCUS are likely to change management. Use of POCUS should not delay arrival to hospital unless the results are critically important as a decision tool for initiating a lifesaving intervention. Improved survival with the use of POCUS has not yet been reported.

#### Airway

Ultrasound may be useful in managing a difficult airway to find cricothyrotomy landmarks or confirm tracheal intubation [228–230].

#### Chest

Prehospital lung ultrasound is highly sensitive for pneumothorax [225, 231]. In one study, 26% of chest decompressions performed by clinical criteria, could have been avoided [227]. Lung ultrasound can also be used to avoid thoracotomies in patients with traumatic circulatory arrest [232]. Ultrasound may also be useful to rule out pericardial effusion with tamponade [233–237] and pneumo- or haemothorax [238].

#### Fractures

In an austere environment, ultrasound performed by an experienced sonographer can accurately diagnose fractrures. POCUS might be used to prevent unnecessary evacuations for suspected extremity fractures that otherwise might need radiographic verification [239]. POCUS can also be used to diagnose pubic symphysis disruption or femur fractures [240], or to elucidate the cause of haemorrhagic shock.

#### Traumatic brain injury

Optic nerve sheath diameter can be measured with POCUS, correlating well with intracerebral pressure in TBI patients [241]. Thus, a pathologic increase of intracerebral pressure can be monitored with POCUS.

#### Abdomen

POCUS can be used to diagnose free fluid from severe blunt trauma to determine the choice of receiving hospital or to guide the diagnostic workup [242]. The Focused Assessment with Sonography for Trauma (FAST) may not be adequate to rule out internal haemorrhage in blunt abdominal trauma [222], but has a reasonable negative predictive value (94%) [243]. FAST may be advantageous in selected cases, but does not improve survival [244], nor is FAST beneficial as part of a triage protocol in mass casualty incidents [245].

#### Technical procedures

POCUS can improve the safety and efficacy of invasive procedures, such as vascular catheterisation [246] and nerve blocks [247].

**Recommendations**: Consider using POCUS in patients with multiple trauma (2B), but do not delay arrival to hospital. Consider ultrasound to manage a difficult airway (2C), to detect pneumo- and haemothorax and pericardial effusion (1A), and to diagnose fractures (2B) and increased intracerebral pressure (2B). Consider performing a FAST exam on patients in shock (1B). Use POCUS for vascular access (1A) and nerve blocks (1A).

### Limitations

Some limitations should be noted. Multiple trauma most often occurs in an urban setting. Most studies address this environment. In mountain areas, high quality evidence regarding treatment of multiple trauma is scarce. This discrepancy was also highlighted by a PubMed literature search on June 23rd 2020. Of 34,055 entries identified with the search term ‘multiple trauma,’ there were only 15 with the search term ‘multiple trauma alpine.’ We expanded the literature search by skimming the reference lists of the articles selected through the PubMed search. We cannot be sure that important studies were not missed with this approach.

A scoping review is not conducted as systematically or in as great a depth as a systematic review, although it still allows development of evidence-based recommendations through a rigorous and transparent approach. In this review, we focused on helicopter-supported mountain rescue missions, mostly because in many developed countries (e.g. Austria > > 98%), as well as in some developing countries, the large majority of multiple trauma patients are rescued by HEMS. Some diagnostic and treatment options presented in this article may be mainly of academic interest (e.g. REBOA, regional anaesthesia, ultrasound). However, our intention was to present an up-to-date approach with all treatment options available worldwide and practiced in different prehospital systems. To date, some treatment options may only be used in a few highly specialized centres, but with technological progress the availability may spread quickly to other systems.

Because there is limited evidence, some of the recommendations rely mainly on expert opinion, at best supported by extrapolation of clinical data to prehospital care in mountain environments.

## Conclusions

Management of patients with multiple trauma in mountain environments can be demanding. Safety of the rescuers and the victim has priority. Use of crABCDE with haemorrhage control first is critical. This should be followed by basic first aid, splinting, immobilisation, analgesia, and insulation. Duration of on-site medical treatment must be balanced against the need for rapid transfer to a trauma centre and should be as short as possible. Reduced on-scene times may be achieved with helicopter rescue. Advanced diagnostics, such as POCUS, may be beneficial. Treatment should be continued during transport.

## Supplementary Information

**Additional file 1.**

**Additional file 2.**

**Additional file 3.**

**Additional file 4.**

## Abbreviations

ABCDE: Airway, Breathing, Circulation, Disability, Exposure
AKI: Acute kidney injury
AVPU: Alert Voice Pain Unresponsive
ARDS: Adult respiratory distress syndrome
bid: twice daily
BP: Blood pressure
crABCDE: critical bleeding, Airway, Breathing Circulation, Disability, Exposure
CNS: Central nervous system
COX: cyclooxygenase
ETCO_2_: End-tidal carbon dioxide
FAST: Focused Assessment with Sonography for Trauma
G: Grams
GCS: Glasgow Coma Scale
HEMS: Helicopter emergency medical service
HES: Hydroxyethyl starch solution
ICAR: International Commission for Alpine Rescue
ICAR MedCom: International Commission for Mountain Emergency Medicine
ICU: Intensive care unit
IM: Intramuscular
IN: Intranasal
IO: intraosseous
ISS: Injury Severity Score
IV: Intravenous
KED: Kendrick Extrication Device
kg: Kilograms
LOE: Level of evidence
MA: Metabolic acidosis
MAP: Mean arterial pressure
mcg: Micrograms
mg: Milligrams
mL: Milliliters
mm Hg: Millimetres of mercury
NaCl: Sodium chloride
NEXUS: National Emergency X-radiography Utilization Study
NSAIDS: Nonsteroidal anti-inflammatory drugs
OTFC: Oral transmucosal fentanyl citrate
PICO: Population Intervention Comparator Outcome
po: by mouth
POCUS: Point of Care Ultrasound
PRICE: Protect the injury, Rest, Ice, Compression, Elevation
qd: daily (dose)
PRBCs: Packed red blood cells
REBOA: Resuscitative Balloon Occlusion of the Aorta
SMR: Spinal motion restriction
SpO_2_: Oxygen saturation by pulse oximetry
SPB: Systolic blood pressure
TBI: Traumatic brain injury
tid: three times daily (dose)
TXA: Tranexamic acid
