# Supplementary material for: Multiple trauma management in mountain environments - a scoping review: Evidence based guidelines of the International Commission for Mountain Emergency Medicine (ICAR MedCom). Intended for physicians and other advanced life support personnel
Source: Scand J Trauma Resusc Emerg Med. 2020 Dec 14;28:117. doi: 10.1186/s13049-020-00790-1 (PMC7737289; doi:10.1186/s13049-020-00790-1)
Supplement: Supplementary file 2 — Additional file 2. [file 13049_2020_790_MOESM2_ESM.docx]

**PICO questions**

**Airway and C-spine**

**Airway Management**

**P**atient Population:  All Patients with airway compromise after multiple trauma in mountainous/alpine terrain (or pre-hospital)

**I**nterventions:  Pre-hospital airway management

**C**omparison: Multiple trauma patients with airway compromise in-hospital

**O**utcome: Mortality, Neurologic adverse outcome, hypoxia, hypercapnia

**Question:**

**In multiple trauma patients in an alpine environment does advanced airway management improve outcome?**

**In multiple trauma in an alpine environment does a specific airway management (e.g. BVM vs. SGA vs. ETI) improve outcome more than others?**

**C-Spine Protection**

**P**atient Population:  All Patients with suspected trauma of the cervical spine after multiple trauma in mountainous/alpine terrain (or pre-hospital)

Sub-population: All patients with severe TBI (traumatic brain injury) and potential trauma of the cervical spine

**I**nterventions:  Pre-hospital airway C-spine protection / immobilization

**C**omparison: Multiple trauma patients with suspected c-spine trauma in-hospital

**O**utcome: Mortality, Neurologic adverse outcome

**Questions:**

**In multiple trauma patients in an alpine environment does C-spine protection improve outcome?**

**In multiple trauma in an alpine environment does a specific C-spine protection improve outcome more than others?**

**Breathing**

**P**atient Population:  All Patients with multiple trauma in mountain terrain with an artificial airway established.

**I**nterventions:  Capnometry or capnography monitoring

**C**omparison: Without capnometry or capnography monitoring

**O**utcome: Mortality, Neurologically intact survival, other morbidities, rate of correct tube position

**Question:**

**In multiple trauma patients in an alpine environment with established artificial airway, does monitoring of end-tidal CO_2_ improve patient safety or outcome?**

**Thoracic trauma**

**P**atient Population:  All Patients with multiple trauma in mountain terrain with thoracic trauma

**I**nterventions:  Pulse oximetry

**C**omparison: Without Pulse oximetry

**O**utcome: Mortality, Neurologically intact survival, other morbidities, sufficient oxygenation

**Question:**

**In multiple trauma patients in an alpine environment with thoracic trauma, does monitoring of peripheral oxygen saturation (pulse oximetry) improve patient safety or outcome?**

**P**atient Population:  All Patients with multiple trauma in mountain terrain with thoracic trauma

Sub-population: Patients with pneumothorax

**I**nterventions:  Transportation with helicopter

**C**omparison: Ground transportation

**O**utcome: Deterioration of the extension of the pneumothorax

**Question:**

**In multiple trauma patients in an alpine environment with thoracic trauma and pneumothorax does transportation by helicopter change the extension of the pneumothorax?**

**P**atient Population:  All Patients with multiple trauma in mountain terrain with thoracic trauma

Sub-population: Patients with pneumothorax

**I**nterventions:  On-scene decompression of pneumothorax.

**C**omparison: No prehospital decompression of pneumothorax.

**O**utcome: Deterioration of the extension of the pneumothorax, Mortality, other morbidities

**Question:**

**In multiple trauma patients in an alpine environment with thoracic trauma and pneumothorax, does on-scene decompression of a pneumothorax reduce the extension of a pneumothorax and improve outcome?**

**P**atient Population:  All Patients with multiple trauma in mountain terrain with thoracic trauma

Sub-population: Patients with pneumothorax

**I**nterventions:  On-scene decompression of pneumothorax.

**C**omparison: Needle vs. tube decompression of pneumothorax.

**O**utcome: Effectiveness of decompression of pneumothorax, complications

**Question:**

**In multiple trauma patients in an alpine environment with thoracic trauma and pneumothorax, is needle or tube decompression more effective to reduce the extension of the pneumothorax and show less complications?**

- Thoracic injury

**P**atient Population:  All Patients with thoracic compromise after multiple trauma in mountainous/alpine terrain (or pre-hospital)

Sub-population: All patients with severe thoracic injuries and potential thoracic compromise

**I**nterventions:  Pre-hospital thoracic injuries management

**C**omparison: Multiple trauma patients with thoracic compromise in-hospital

**O**utcome: Mortality, Neurologic adverse outcome, hypoxia, hypercapnia

**Question**:

Does thoracic injuries management in patients suffering from acute or potential airway compromise after multiple trauma in alpine environment differ from in-hospital management?

Do these patient groups differ in the amount of hypoxia / hypercapnia, neurologic adverse outcome or mortality?

**Circulation**

- Non-Pharmacological Bleeding Control Management

**P**atient Population:  All Patients with external and/or suspected internal bleeding after multiple trauma in mountainous/alpine terrain (or pre-hospital)

Sub-population: All patients with potential internal bleeding

**I**nterventions:  Pre-hospital control bleeding management

**C**omparison: Multiple trauma patients with bleeding in-hospital

**O**utcome: Mortality, Neurologic adverse outcome, shock

**Question**:

Does control bleeding management in patients suffering from acute or potential bleeding after multiple trauma in alpine environment differ from in-hospital management?

Do these patient groups differ in the amount of shock, neurologic adverse outcome or mortality?

**Disability**

**1) Primary Brain Injury Prevention.**

*Patient Population:  All Patients with Traumatic Brain Injury in Mountainous /Alpine Wilderness terrain (or pre-hospital)*

*Interventions:  Use of Helmets, role in prevention of TBI*

*Comparison: Incidence and severity of TBI in non- Helmeted victims*

*Outcomes: Mortality, Neurologic adverse outcome*

**2) Airway Management in TBI.**

*Patient Population:  All Patents with Traumatic Brain Injury in Mountainous/Alpine/Wilderness terrain (or pre-hospital)*

*Interventions:  Pre Hospital Airway Management with endotracheal intubation*

*Comparison: TBI patients basic airway/ no management*

*Outcomes: Mortality, Neurologic adverse outcome*

**3) Oxygenation and Ventilation.**

Patient Population:  All Patients with Traumatic Brain Injury in Mountainous/Alpine /Wilderness terrain (or pre-hospital)

Interventions:  Pre Hospital Ventilation management (Oxygenation, CO_2_)

Comparison: TBI patients with Hypoxia - Hypercapnia

Outcomes: Mortality, Neurologic adverse outcome

**4) Arterial Hypotension.**

*Patient Population:  All Patients with Traumatic Brain Injury in Mountainous/Alpine / Wilderness terrain ( or pre-hospital )*

*Interventions:  Pre Hospital management of hypotension*

*Comparison: outcomes of patients with TBI and no hypotension*

*Outcomes: Mortality, Neurologic adverse outcome*

**5) Methods to decrease ICP.**

*Patient Population:  All Patients with Traumatic Brain Injury in Mountainous/Alpine / Wilderness terrain ( or pre-hospital*

Interventions: Pre Hospital Drugs/interventions to manage ICP

- Head elevation
- Mannitol
- Hypertonic Saline

Comparison: TBI patients with elevated ICP

Outcomes: Mortality, Neurologic adverse outcome

**6) Methods to decrease hematoma growth.**

*Patient Population:  All Patients with Traumatic Brain Injury in Mountainous/Apine / Wilderness terrain (or pre-hospital)*

Interventions:  early use/prehospital use of Tranexamic acid

Comparison: No treatment

Outcomes: Mortality, Neurologic adverse outcome

**7) Hypothermia and TBI**

*Patient Population:  All Patients with Traumatic Brain Injury in Mountainous/Alpine / Wilderness terrain (or pre-hospital)*

Interventions:  early induced therapeutic hypothermia

Comparison: no treatment

Outcomes: Mortality, Neurologic adverse outcome

**8) Methods of rescue and transportation.**

*Patient Population:  All Patients with Traumatic Brain Injury in Mountainous/Alpine / Wilderness terrain ( or pre-hospital )*

*Interventions:  Pre Hospital transport modality, pre- hospital staff resources and time to definitive care*

*Outcomes: Mortality, Neurologic outcome*

**Spinal injury**

***1)The need for motion control (immobilisation)***

*Patient Population:  All Patents with potential spinal Injury in Mountainous/Alpine/ Wilderness terrain (or pre-hospital)*

*Interventions: Spinal motion control (immobilisation)*

*Comparison: Spinal injury patients without spinal motion control*

*Outcomes: Mortality, Neurologic adverse outcome*

***2) Techniques for motion control***

*Patient Population:  All Patents with potential spinal Injury in Mountainous/Alpine /Wilderness terrain (or pre-hospital)*

*Interventions: Spine board, collar, head blocks, vacuum mattress*

*Comparison: Compared methods*

*Outcomes: Mortality, Neurologic adverse outcome, morbidity.*

***3)The need for a cervical collar****.*

*Patient Population:  All Patents with potential spinal Injury in Mountainous/Alpine /Wilderness terrain (or pre-hospital)*

*Interventions:, cervical collar*

*Comparison: no collar or alternative motion control*

*Outcomes: Mortality, Neurologic adverse outcome, morbidity.*

**4) Moving and Handling patients with suspected spinal injury.**

*Patient Population:  All Patents with potential spinal Injury in Mountainous/Alpine/ Wilderness terrain (or pre-hospital)*

*Interventions: Log roll*

*Comparison: scoop stretcher, vertical lift*

*Outcomes: Mortality, Neurologic adverse outcome, morbidity.*

**Environment and exposure**

*PICO: In multiple injured trauma patients in an alpine environment, what is the reliability of the exposure phase of the primary assessment and physical exam to detect traumatic injuries?*

*PICO: In multiple injured trauma patients in an alpine environment, is the information obtained during the exposure phase of the primary assessment and physical exam of significant contribution to aid on field evacuation decisions?*

*PICO: In multiple injured trauma patients in alpine environments what is the associated risk of further exposure to the environment during the exposure phase of the primary assessment and physical exam*

**First aid, splinting and immobilization**

*PICO 1: Patient Population. All Patients with closed extremity bone fractures in Mountainous /alpine terrain (or pre-hospital)*

*Interventions: Use of extremity splints*

*Comparison: no splint or different types of splints (vacuum, Sam, air, improvised)*

*Outcomes: Morbidity (pain control. chronic sequela), Mortality*

*PICO: Patient Population. All Patients with closed extremity femur  fractures in Mountainous /alpine terrain (or pre-hospital )*

*Interventions:  Use of femur traction splints*

*Comparison: no splint or splint without traction splints (vacuum, Sam, air, improvised)*

*Outcomes: Morbidity (pain control, chronic sequelae), Mortality*

**Analgesia**

*PICO: In multiply injured trauma patients in an alpine environment, what non-pharmacologic techniques reduce pain?*

*PICO: In multiply injured trauma patients in an alpine environment, does splinting extremity injuries assist with pain?*

*PICO: In unstable trauma patients in an alpine environment, do analgesic medications worsen hemodynamics? Do analgesic medications worsen respiratory function or increase need for ventilatory interventions?*

**Temperature Management**

Question 1

Population: patients with multiple trauma in mountain environments or pre-hospital in rural and remote areas

Intervention: pre-hospital temperature measurement for detection of hypothermia

Comparison: none

Outcomes: decision of triage, morbidity and mortality of hypothermia + trauma, measurement reliability, field applications

Question 2

Population: patients with multiple trauma in mountain environments or pre-hospital in rural and remote areas

Intervention: pre-hospital heat loss prevention

Comparison: none

Outcomes: methods, morbidity and mortality of hypothermia + trauma, field applications

Question 3

Population: patients with multiple trauma in mountain environments or pre-hospital in rural and remote areas

Intervention: pre-hospital re-warming

Comparison: none

Outcomes: methods, morbidity and mortality of hypothermia + trauma, field applications

**Triage and hospital selection**

**Hospital selection**

**P**atient Population:  All Patients with multiple trauma in mountain terrain.

Sub-population: All patients with TBI

**I**nterventions:  Hospital selection (trauma center vs nontrauma center)

**C**omparison: Urban patients with multiple trauma

**O**utcome: Mortality, Neurologically intact survival, other morbidities

**Question:**

**Does selection of hospital differ in patients with multiple trauma (with and without TBI) in mountain terrain and urban settings.**

**Do outcomes of patients with multiple trauma in mountain terrain, differ between those transported to a trauma center and those transported to non-trauma center**

**Transport**

**Helicopter transport**

**P**atient Population:  All Patients with multiple trauma in mountain terrain.

Sub-population: All patients with TBI

**I**nterventions:  Helicopter transport

**C**omparison: Helicopter vs terrestrial transport

**O**utcome: Mortality, Neurologically intact survival, other morbidities

**Question:**

**Do the outcomes of multiple trauma patients in mountain terrain transported by helicopter differ from the outcome of those transported by terrestrial methods.**

**Prolonged field care**

**P**atient Population:  All Patients with multiple trauma in mountain terrain.

Sub-population: All patients with TBI

**I**nterventions:  Prolonged field care

**C**omparison: Prolonged field care versus early transport

**O**utcome: Mortality, Neurologically intact survival, other morbidities

**Question:**

**Do the outcomes of multiple trauma patients in mountain terrain with prolonged field care differ from those transported by air or terrestrial transport?**

**Ultrasound**

PICO: *In multiply injured trauma patients in an alpine environment, does POCUS help in diagnostic and triage and hospital destination?*

PICO: *In multiply injured trauma patients in an alpine environment, is POCUS helpful in chest trauma assessment?*

PICO: *In multiply injured trauma patients in an alpine environment, is POCUS helpful*

*In abdominal trauma assessment?*

PICO: *In multiply injured trauma patients in an alpine environment, is POCUS helpful in head trauma assessment?*

PICO: *In multiply injured trauma patients in an alpine environment, is POCUS helpful and safe for technical procedure?*
