# Supplementary material for: Multiple trauma management in mountain environments - a scoping review: Evidence based guidelines of the International Commission for Mountain Emergency Medicine (ICAR MedCom). Intended for physicians and other advanced life support personnel
Source: Scand J Trauma Resusc Emerg Med. 2020 Dec 14;28:117. doi: 10.1186/s13049-020-00790-1 (PMC7737289; doi:10.1186/s13049-020-00790-1)
Supplement: Supplementary file 3 — Additional file 3. [file 13049_2020_790_MOESM3_ESM.docx]

**LITERATURE SEARCH**

**TEMPLATE**

**PubMed-Search (updated to 30^th^ September 2019)**

**Search hits**

1. "Multiple trauma" AND "Alpine" AND "Circulation" 1

2. "Multiple trauma" AND "Mountain" AND "Circulation" 2

3. "Multiple trauma" AND "Alpine" AND "Shock" 1

4. "Multiple trauma" AND "Mountain" AND "Shock" 2

5. "Multiple Trauma" AND "Alpine" AND "Hypovolemic" 0

6. "Multiple Trauma" AND "Mountain" AND "Hypovolemic" 0

7. "Multiple Trauma" AND "Alpine" AND "Crystalloids" 0

8. "Multiple Trauma" AND "Mountain" AND "Crystalloids" 0

9. "Multiple Trauma" AND "Alpine" AND "Colloids" 0

10. "Multiple Trauma" AND "Mountain" AND "Colloids" 0

11. "Multiple Trauma" AND "Alpine" AND "Fluids" 1

12. "Multiple Trauma" AND "Mountain" AND "Fluids" 0

13. "Multiple trauma" AND "Alpine" AND "Volume" 0

14. "Multiple trauma" AND "Mountain" AND "Volume" 0

15. "Multiple Trauma" AND "Alpine" AND "Hypovolemic" 0

16. "Multiple Trauma" AND "Mountain" AND "Hypovolemic" 0

17. “Multiple trauma” AND “Wilderness” 11

18. “Trauma” AND “Wilderness” 742

19. “Polytrauma” AND “Mountain” AND” Circulation” 3

20. “Polytrauma” AND “Alpine” AND “Circulation” 1

21. “Pre-hospital” AND “Wilderness” 64

22. “Austere” AND “Tourniquets” 10

**Rescuer safety**

Safety and risk

**PubMed-Search (from 30th September 2020)** (Guenther)

**Search hits**

1. “multiple trauma” AND “mountain” AND “safety” 0
2. “multiple trauma” AND “alpine” AND “safety” 1
3. “polytrauma” AND “mountain” AND “safety” 0
4. “polytrauma” AND “alpine” AND “safety” 1
5. “multiple trauma” AND “mountain” AND “risk” 3
6. “multiple trauma” AND “alpine” AND “risk” 3
7. "polytrauma" AND "mountain" AND "risk" 1
8. "polytrauma" AND "alpine" AND "risk" 2
9. “multiple trauma” AND “mountain rescue” 2
10. “multiple trauma” AND “alpine rescue” 1
11. “polytrauma” AND “mountain rescue” 2
12. “polytrauma” AND “alpine rescue” 0
13. “multiple trauma” AND “mountain” AND “hazard” 0
14. “multiple trauma” AND “alpine” AND “hazard” 0
15. “polytrauma” AND “mountain” AND “hazard” 0
16. “polytrauma” AND “alpine” AND “hazard” 0
17. “multiple trauma” AND "mountain" 16
18. “multiple trauma” AND "alpine” 9
19. “polytrauma” AND "mountain" 4
20. “polytrauma” AND "alpine" 3
21. “multiple trauma” AND "wilderness" 8
22. “polytrauma” AND "wilderness" 3
23. “mountain rescue” AND “evacuation” 14
24. “alpine rescue” AND “evacuation” 2

**Airway and C-spine**

Up to 30th September 2019

1. PubMed:

1. trauma airway management prehospital 727
2. trauma cervical spine prehospital 187
3. immobilization prehospital 299

**Breathing**

**PubMed-Search (until 30th September 2019)** (Guenther)

**Search hits**

1. “intubation” AND "end-tidal carbon dioxide" AND "prehospital" 32
2. “intubation” AND "end-tidal carbon dioxide" AND "mountain" 0
3. “ventilation” AND "end-tidal carbon dioxide" AND "prehospital" 26
4. “ventilation” AND "end-tidal carbon dioxide" AND "mountain" 3
5. “multiple trauma” AND “oxygen” 304
6. "multiple trauma" AND "oxygen" AND "mountain" 0
7. "multiple trauma" AND "oxygen" AND "alpine" 0

**Thoracic trauma**

**PubMed-Search (until 30th September 2019)** (Guenther)

**Search hits**

"thoracic trauma" AND "pulse oximetry" 4

"thoracic trauma" AND "pulse oximetry" AND “mountain” 0

"thoracic trauma" AND "ventilation" AND "analgesia" 17

"thoracic trauma" AND "ventilation" AND "analgesia" AND "mountain" 0

“thoracic trauma” AND “oxygen” 55

“thoracic trauma” AND “oxygen” AND “mountain” 0

“pneumothorax” AND “helicopter” 37

“pneumothorax” AND “helicopter” AND “mountain” 0

"pneumothorax" AND "helicopter" AND "multiple trauma" 5

"tension pneumothorax" AND "decompression" AND "prehospital" 32

"tension pneumothorax" AND "decompression" AND "mountain" 0

"tension pneumothorax" AND "decompression" AND "alpine" 0

"tension pneumothorax" AND "thoracostomy" AND "prehospital" 29

"tension pneumothorax" AND "thoracostomy" AND "mountain" 0

"tension pneumothorax" AND "thoracostomy" AND "alpine" 0

Thoracic injury

**PubMed-Search (Until 30^th^ September 2019)**

**Search hits**

1. “multiple trauma” AND “thoracic trauma” AND “alpine” 1
2. “multiple trauma” AND “thoracic injuries” AND “alpine” 1
3. “multiple trauma” AND “thoracic trauma” AND “mountain” 1
4. “multiple trauma” AND “thoracic injuries” AND “mountain” 1
5. “multiple trauma” AND “thoracic trauma” AND “wilderness” 0
6. “multiple trauma” AND “thoracic injuries” AND “wilderness” 0
7. “multiple trauma” AND “thoracic trauma” AND “austere2 2
8. “multiple trauma” AND “thoracic injuries” AND “austere2 2
9. “poly trauma” AND “thoracic trauma” AND “alpine” 0
10. “poly trauma” AND “thoracic trauma” AND “mountain” 0
11. “poly trauma” AND “thoracic trauma” AND “wilderness” 0
12. “poly trauma” AND “thoracic trauma” AND “austere” 0
13. “multiple trauma” AND “thoracic injuries” AND “out of hospital” 105
14. “multiple trauma” AND “thoracic trauma” AND “out of hospital 122
15. “multiple trauma” AND “thoracic injuries” AND “pre-hospital” 20
16. “multiple trauma” AND “thoracic trauma” AND “pre-hospital 25
17. “multiple trauma” AND “chest injuries” AND “out of hospital” 140
18. “multiple trauma” AND “chest injurie” AND “pre-hospital 30
19. “multiple trauma” AND “chest trauma” AND “out of hospital” 160
20. “multiple trauma” AND “chest trauma” AND “pre-hospital” 34
21. “thoracic trauma” AND “alpine” 20
22. “thoracic injuries” AND “alpine” 15
23. “thoracic trauma” AND “wilderness” 17
24. “thoracic injuries” AND “wilderness” 15
25. “thoracic trauma” AND “mountain” 37
26. “thoracic injuries” AND “mountain” 31
27. “thoracic trauma” AND “austere” 20
28. “thoracic injuries” AND “austere” 21
29. “thoracic injuries” AND “out of hospital” 775
30. “thoracic injuries” AND “pre-hospital” 458
31. “thoracostomy” AND “alpine” 0
32. “thoracostomy” AND “mountain” 1
33. “thoracostomy” AND “wilderness” 5
34. “thoracostomy” AND “austere 6
35. “thoracostomy” AND “out of hospital” 100
36. “thoracostomy” AND “pre-hospital” 11
37. “needle thoracostomy” AND “alpine” 0
38. “needle thoracostomy” AND “mountain” 1
39. “needle thoracostomy” AND “wilderness” 5
40. “needle thoracostomy” AND “austere” 6
41. “needle thoracostomy” AND “out of hospital” 100
42. “needle thoracostomy” AND “pre-hospital” 11
43. “tension pneumothorax ” AND “alpine” 2
44. “tension pneumothorax ” AND “mountain” 24
45. “tension pneumothorax ” AND “wilderness” 12
46. “tension pneumothorax ” AND “austere” 11
47. “tension pneumothorax ” AND “out of hospital” 642
48. “tension pneumothorax ” AND “pre-hospital” 25
49. “traumatic open pneumothorax” 49
50. “traumatic open pneumothorax” AND “alpine” 0
51. “traumatic open pneumothorax” AND “mountain” 0
52. “traumatic open pneumothorax” AND “wilderness” 1
53. “traumatic open pneumothorax” AND “austere” 1
54. “open pneumothorax” AND “out of hospital” 41
55. “open pneumothorax” AND “pre-hospital” 2
56. “pneumothorax” AND “altitude” 52
57. “open pneumothorax” AND “altitude” 0
58. “traumatic pneumothorax” AND “altitude” 4
59. “traumatic tension pneumothorax” AND “altitude” 4
60. “massive hemothorax” AND “alpine” 0
61. “massive hemothorax” AND “mountain” 0
62. “massive hemothorax” AND “wilderness” 1
63. “massive hemothorax” AND “austere” 0
64. “massive hemothorax” AND “pre-hospital” 3
65. “massive hemothorax” AND “out of hospital” 19
66. “massive hemothorax” AND “wilderness” 1
67. “massive hemothorax” AND “polytrauma” 38
68. “flail chest ” AND “alpine” 0
69. “flail chest ” AND “mountain” 0
70. “flail chest ” AND “wilderness” 0
71. “flail chest ” AND “austere” 0
72. “flail chest ” AND “out of hospital” 26
73. “flail chest ” AND “pre-hospital” 2
74. “chest decompression” AND “alpine” 0
75. “chest decompression” AND “mountain” 2
76. “chest decompression” AND “wilderness” 1
77. “chest decompression” AND “austere” 1
78. “chest decompression” AND “out of hospital” 65
79. “chest decompression” AND “pre-hospital” 14
80. “open pneumothorax” AND “dressing” 12
81. “open pneumothorax” AND “dressing” AND “alpine” 0
82. “open pneumothorax” AND “dressing” AND “mountain” 12
83. “open pneumothorax” AND “dressing” AND “wilderness” 0

**Circulation**

**- Non-Pharmacological bleeding control**

**To September 30 2019**

**Search hits**

1. “multiple trauma” AND “external bleeding control” AND “prehospital” 3
2. “multiple trauma” AND “external bleeding control” AND “out of hospital” ５
3. “multiple trauma” AND “control bleeding” AND “out of hospital” 31
4. “multiple trauma” AND “control bleeding” AND “prehospital” 46
5. “multiple trauma” AND “non-pharmacological control bleeding” AND “prehospital” 0
6. “multiple trauma” AND “non-pharmacological control bleeding” AND “out of hospital“ 0
7. “multiple trauma” AND “compressible bleeding” AND “prehospital” 2
8. “multiple trauma” AND “compressible bleeding” AND “out of hospital” 0
9. “multiple trauma” AND “compressible bleeding” AND “alpine” 0
10. “multiple trauma” AND “compressible bleeding” AND “mountain” 0
11. “multiple trauma” AND “compressible bleeding” AND “wilderness” 0
12. “multiple trauma” AND “compressible bleeding” AND “austere” 0
13. “multiple trauma” AND “compressible hemorrhage” AND “prehospital” 2
14. “multiple trauma” AND “compressible hemorrhage” AND “out of hospital“ 1
15. “multiple trauma” AND “compressible hemorrhage” AND “alpine” 0
16. “multiple trauma” AND “compressible hemorrhage” AND “mountain” 0
17. “multiple trauma” AND “compressible hemorrhage” AND “wilderness” 0
18. “multiple trauma” AND “compressible hemorrhage” AND “austere” 0
19. “multiple trauma” AND “hemorrhage control” AND “out of hospital” 27
20. “multiple trauma” AND “hemorrhage control” AND “prehospital” 0
21. “multiple trauma” AND “hemorrhage control” AND “alpine” 0
22. “multiple trauma” AND “hemorrhage control” AND “mountain” 0
23. “multiple trauma” AND “hemorrhage control” AND “wilderness” 0
24. “multiple trauma” AND “hemorrhage control” AND “austere” 4
25. “multiple trauma” AND “external hemorrhage” AND “out of hospital“ 7
26. “multiple trauma” AND “external hemorrhage” AND “prehospital” 5
27. “hemorrhage control” AND “blunt trauma” AND “out of hospital” 23
28. “hemorrhage control” AND “blunt trauma” AND “prehospital” 32
29. “hemorrhage control” AND “blunt trauma” AND “alpine” 0
30. “hemorrhage control” AND “blunt trauma” AND “mountain” 0
31. “hemorrhage control” AND “blunt trauma” AND “wilderness” 0
32. “hemorrhage control” AND “blunt trauma” AND “austere” 3
33. “Tourniquet” AND “out of hospital“ 181
34. “Tourniquet” AND “pre-hospital” 26
35. “Tourniquet” AND “alpine” 0
36. “Tourniquet” AND “mountain” 2
37. “Tourniquet” AND “wilderness” 27
38. “Tourniquet” AND “austere” 14
39. “Tourniquet Conversion” 38
40. “Tourniquet Conversion” AND “out of hospital” 1
41. “Tourniquet Conversion” AND “pre-hospital” 0
42. “Tourniquet Conversion” AND “alpine” 0
43. “Tourniquet Conversion” AND “mountain” 0
44. “Tourniquet Conversion” AND “wilderness” 0
45. “Tourniquet Conversion” AND “austere” 0
46. “multiple trauma” AND “pelvic binder” 9
47. “multiple trauma” AND “pelvic binder” AND “alpine” 0
48. “multiple trauma” AND “pelvic binder” AND “mountain“ 0
49. “multiple trauma” AND “pelvic binder” AND “wilderness” 0
50. “multiple trauma” AND “pelvic binder” AND “austere” 0
51. “multiple trauma” AND “pelvic binder” AND “out of hospital” 1
52. “multiple trauma” AND “pelvic binder” AND “pre-hospital” 1
53. “multiple trauma” AND “pelvic fracture” AND “alpine” 0
54. “multiple trauma” AND “pelvic fracture” AND “mountain” 2
55. “multiple trauma” AND “pelvic fracture” AND “wilderness” 0
56. “multiple trauma” AND “pelvic fracture” AND “austere” 0
57. “pelvic binder” 112
58. “pelvic binder” AND “out of hospital” 9
59. “pelvic binder” AND “pre-hospital” 3
60. “pelvic binder” AND “austere” 2
61. “pelvic external fixation” AND “ out of hospital” 23
62. “pelvic external fixation” AND “ pre-hospital“ 2
63. “pelvic external fixation” AND “ alpine” 0
64. “pelvic external fixation” AND “ mountain” 0
65. “pelvic external fixation” AND “ wilderness” 0
66. “pelvic external fixation” AND “austere” 3
67. “pelvic fracture” AND “hemorrhage control” 323
68. “pelvic fracture” AND “hemorrhage control” AND “out of hospital” 14
69. “pelvic fracture” AND “hemorrhage control” AND “pre-hospital” 2
70. “pelvic fracture” AND “hemorrhage control” AND “alpine” 0
71. “pelvic fracture” AND “hemorrhage control” AND “mountain” 0
72. “pelvic fracture” AND “hemorrhage control” AND “wilderness” 0
73. “pelvic fracture” AND “hemorrhage control” AND “austere“ 1
74. “pelvic fracture” AND “bleeding control” 359
75. “pelvic fracture” AND “bleeding control” AND “alpine” 0
76. “pelvic fracture” AND “bleeding control” AND “mountain” 0
77. “pelvic fracture” AND “bleeding control” AND “wilderness” 0
78. “pelvic fracture” AND “bleeding control” AND “austere” 3
79. “pelvic fracture” AND “bleeding control” AND “out of hospital” 17
80. “pelvic fracture” AND “bleeding control” AND “pre-hospital” 3

**Disability**

**1) Primary Brain Injury Prevention.**

**Search**

1. Helmet AND alpine= 40
2. Helmet AND mountain = 31
3. Helmet AND mountain AND mortality = 2
4. Helmet AND mountain AND morbidity = 2
5. Helmet AND mountain AND outcome= 5
6. Helmet AND alpine AND outcome = 5
7. Multiple trauma AND traumatic brain injury AND prevention = 164

**2) Airway Management in TBI.**

**Search**

1. Multiple trauma AND traumatic brain injury AND Airway = 18
2. Multiple trauma AND traumatic brain injury AND Airway AND mortality= 3
3. Multiple trauma AND traumatic brain injury AND Airway AND morbidity= 4
4. Multiple trauma AND traumatic brain injury AND Airway AND neurological outcome = 1
5. Multiple trauma AND traumatic brain injury AND Endotracheal intubation= 19
6. Multiple trauma AND traumatic brain injury AND Endotracheal intubation AND mortality = 10
7. Multiple trauma AND traumatic brain injury AND Endotracheal intubation AND morbidity = 7
8. Multiple trauma AND traumatic brain injury AND Endotracheal intubation AND neurological outcome = 0
9. Multiple trauma AND traumatic brain injury AND Airway AND alpine / mountain/ wilderness = 0

**3) Oxygenation and Ventilation.**

**Search**

1. Multiple trauma AND traumatic brain injury AND oxygenation = 45
2. Traumatic brain injury AND hypoxia AND mortality = 211
3. Multiple trauma AND traumatic brain injury AND ventilation = 63
4. Multiple trauma AND traumatic brain injury AND ventilation AND neurological outcome = 3
5. Multiple trauma AND traumatic brain injury AND ventilation AND mortality= 26
6. Multiple trauma AND traumatic brain injury AND ventilation AND morbidity=40
7. Traumatic brain injury AND hypercapnia AND mortality = 23
8. Traumatic brain injury AND hypercapnia AND neurological outcome = 17
9. Multiple trauma AND traumatic brain injury AND ventilation AND neurological outcome = 3
10. Multiple trauma AND traumatic brain injury AND ventilation AND alpine / mountain/ wilderness = 0
11. Multiple trauma AND traumatic brain injury AND airway AND alpine / mountain/ wilderness = 0

**4) Arterial Hypotension.**

**Search**

Multiple trauma AND traumatic brain injury AND hypotension AND mortality = 36

Multiple trauma AND traumatic brain injury AND hypotension AND morbidity = 37

Multiple trauma AND traumatic brain injury AND hypotension AND neruologcal outcome = 4

Multiple trauma AND traumatic brain injury AND hypotension AND alpine /mountain / wilderness = 0

Multiple trauma AND traumatic brain injury AND hypotension AND pre hospital care=4

Multiple trauma AND traumatic brain injury AND hypotension AND emergency medical service = 13

**5) Methods to decrease ICP.**

**Search**

Multiple trauma AND traumatic brain injury AND head elevation = 8

Multiple trauma AND traumatic brain injury AND head elevation AND Alpine/ wilderness/ mountain = 0

Traumatic brain injury AND head elevation = 141

Traumatic brain injury AND head elevation AND mortality = 22

Traumatic brain injury AND head elevation AND morbidity = 27

Traumatic brain injury AND head elevation AND pre hospital care = 0

Traumatic brain injury AND head elevation AND emergency medical service = 5

Multiple trauma AND traumatic brain injury AND hypertonic saline = 26

Traumatic brain injury AND hypertonic saline = 293

Traumatic brain injury AND hypertonic saline AND mortality = 92

Traumatic brain injury AND hypertonic saline AND morbidity = 62

Traumatic brain injury AND hypertonic saline AND neurological outcome = 25

Traumatic brain injury AND hypertonic saline AND Alpine / Wilderness/Mountain=0

Traumatic brain injury AND manitol AND mortality = 76

Traumatic brain injury AND manitiol AND neurological outcome = 80

**6) Methods to decrease hematoma growth.**

**Search**

Traumatic brain injury tranexamic acid = 60

Traumatic brain injury AND hypertonic saline AND mortality = 92

Traumatic brain injury AND hypertonic saline AND morbidity = 62

Traumatic brain injury AND hypertonic saline AND Alpine/Wilderness/ Mountain =0

Traumatic brain injury AND hypertonic saline AND multiple trauma = 4

Traumatic brain injury AND hypertonic saline AND polytrauma = 13

**7) Hypothermia and TBI**

**Search**

Multiple trauma AND traumatic brain injury AND hypothermia = 39

1. Poly trauma AND traumatic brain injury AND hypothermia = 3
2. Traumatic brain injury AND hypothermia = 854
3. Traumatic brain injury AND hypothermia AND mortality = 155
4. Traumatic brain injury AND hypothermia AND morbidity = 124
5. Traumatic brain injury AND hypothermia AND neurological outcome = 368

**8) Methods of rescue and transportation.**

**Search**

Traumatic brain injury AND pre hospital time = 275

Traumatic brain injury AND pre hospital time AND mortality = 54

Traumatic brain injury AND time AND transport AND mortality = 57

Traumatic brain injury AND golden hour = 5

Traumatic brain injury AND air transport = 45

Traumatic brain injury AND air transport AND mortality = 23

Traumatic brain injury AND helicopter = 93

Traumatic brain injury AND on scene time = 69

**Spinal injury**

Multiple trauma AND spinal injury AND Alpine = 3

Multiple trauma AND spinal injury AND Mountain = 1

Multiple trauma AND spinal injury AND Wilderness = 2

Multiple trauma AND spinal injury AND immobilisation = 80

Multiple trauma AND spinal injury AND immobilisation AND mortality = 10

Multiple trauma AND spinal injury AND immobilisation AND morbidity = 17

Multiple trauma AND spinal injury AND immobilisation AND neurological outcome = 7

Spinal injury AND immobilisation AND prehospital = 49

Spinal injury AND immobilisation AND emergency medical service = 329

Spinal injury AND immobilisation AND cervical collar = 233

Spinal injury AND immobilisation AND spine board = 65

Spinal injury AND immobilisation AND scoop stretcher = 7

Spinal injury AND immobilisation AND manual in line stabilisation = 21

Spinal injury AND immobilisation AND vacuum mattress = 21

Spinal injury AND immobilisation AND head blocks = 6

**Environment and exposure**

Last search 30/09/2019

**Search hits**

1. "Multiple trauma" AND "Alpine" AND "Exposure" 0
2. "Multiple trauma" AND "Mountain" AND "Exposure" 4
3. "Multiple trauma" AND "prehospital" AND "Exposure" 17
4. "Multiple trauma" AND "ems" AND "Exposure" 4
5. "Multiple trauma" AND "Alpine" AND "physical exam" 0
6. "Multiple trauma" AND "Mountain" AND "physical exam" 4
7. "Multiple Trauma" AND "prehospital" AND "physical exam" 46
8. "Multiple trauma" AND "ems" AND "physical exam" 7
9. “Prehospital” AND “physical exam” 12
10. “Prehospital” AND “physical assessment” (filter=humans) 918
11. “Prehospital” [Title] AND “physical assessment” [Title] 1
12. Traumatic injuries AND physical exam (filter=humans) 11

**First aid, splinting and immobilization**

First aid pre-hospital 177

Splinting pre-hospital 17

Immobilization pre-hospital 69

**Analgesia**

Non-pharmacologic analgesia

Last search 30 September 2019

1. „Analgesia“ AND „pre-hospital“ AND „non-pharmacologic“ 0
2. „Analgesia“ AND „trauma“ AND „non-pharmacologic“ 5

**Temperature Managment**

| Search term | Date | Filters | Results |
| --- | --- | --- | --- |
| trauma AND pre-hospital AND temperature measurement | 5/13 | none | 7 (?6) |
| pre-hospital AND temperature measurement | 5/13 | none | 15 |
| trauma AND mountain AND temperature measurement | proposal | none | 13 to be screened |
| trauma AND out-of-hospital AND temperature measurement | proposal | none | 1 to be screened |
| trauma AND pre-hospital AND temperature management | proposal | none | 23 to be screened |
| trauma AND out-of-hospital AND temperature management | proposal | none | 46 to be screened |
| trauma AND mountain AND temperature management | proposal | none | 13 to be screened |
| trauma AND pre-hospital AND hypothermia | proposal | none | 51 to be screened |
| trauma AND out-of-hospital AND hypothermia | proposal | none | 135 to be screened |
| trauma AND mountain AND hypothermia | proposal | none | 74 to be screened |
| trauma AND pre-hospital AND hypothermia AND rewarming | proposal | none | 12 to be screened |
| trauma AND mountain AND hypothermia AND rewarming | proposal | none | 22 to be screened |
| trauma AND out-of-hospital AND hypothermia AND rewarming | proposal | none | 23 to be screened |
|  |  |  |  |
| ((core temperature[Title/Abstract]) AND measurement[Title/Abstract]) AND field[Title/Abstract] | 5/13 | humans | 22 |

**Hospital selection and transport**

PubMed search 30^th^ Sept 2019

**Search hits**

1. "multiple trauma" AND "mountain" AND "trauma center” 2
2. "multiple trauma" AND "mountain" AND "transport” 1
3. "multiple trauma" AND "mountain" AND "transport to trauma center” 0
4. "multiple trauma" AND "mountain" AND "evacuation" 1
5. "polytrauma" AND "mountain" AND "trauma center” 1
6. "polytrauma" AND "mountain" AND "transport” 0
7. "polytrauma" AND "mountain" AND "evacuation” 0
8. "multiple trauma" AND "transport to trauma center” 133
9. "multiple trauma" AND "transport to trauma center” AND “survival” 41
10. "multiple trauma" AND "transport to trauma center” AND “outcome” 51
11. "multiple trauma" AND "mountain" AND "helicopter" 3
12. "multiple trauma" AND "helicopter" AND "outcome" 57
13. "multiple trauma" AND “mountain” AND "helicopter" AND "outcome" 1
14. "multiple trauma" AND "mountain" AND "helicopter" AND "trauma center" 1

**Ultrasound (Final update)**

30^th^ Sept 2019

Ultrasound trauma prehospital 341 hits
