# Supplementary material for: Multiple trauma management in mountain environments - a scoping review: Evidence based guidelines of the International Commission for Mountain Emergency Medicine (ICAR MedCom). Intended for physicians and other advanced life support personnel
Source: Scand J Trauma Resusc Emerg Med. 2020 Dec 14;28:117. doi: 10.1186/s13049-020-00790-1 (PMC7737289; doi:10.1186/s13049-020-00790-1)
Supplement: Supplementary file 4 — Additional file 4. [file 13049_2020_790_MOESM4_ESM.docx]

| The **ICAR MEDCOM**  Commission for Mountain Medicine  of the International Commission for Alpine Rescue  [www.alpine-rescue.org](http://www.alpine-rescue.org) | 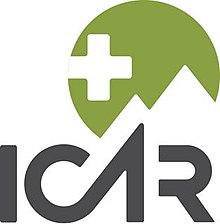 |
| --- | --- |

**ICAR MEDCOM RECOMMENDATION**

| **Nr.** | MED-REC-2020-001 |
| --- | --- |
| **Version** | 1.0 |
| **Title** | **Multiple Trauma Management in Mountain Environments** - A scoping review |
| **Author(s)** | Sumann Guenther, Moens Didier, Brink Bruce, Brodmann-Maeder Monika, Greene Mike, Jacob Mathias, Koirala Pranawa, Zafren Ken, Ayala Marin, Musi Martin, Oshiro Kazue, Sheets Alison, Strapazzon Giacomo, Macias Darryl, Paal Peter |
| **Responsible person** | Paal Peter |
| **Originally based on** | N/A |
| **Date of publication** | XX/XX/2020 |
| **Valid Until** | (at most 5 years later) |

**1. Background**

Multiple trauma in the mountains may be associated with increased morbidity and mortality compared to urban environments. The objective of the paper is to provide evidence-based guidance to assist the rescuer in multiple trauma management in mountain environments and to present an algorithm for the management of multiple trauma in mountainous terrain.

**2. Recommendations**

| **Nr.** | **Recommendation** | **Grade** |
| --- | --- | --- |
|  | **Situation awareness and Safety** |  |
| 1 | Consider terrain, weather, transport conditions and limited resources when treating a multiple trauma patient in the mountains. | 1C |
| 2 | On-scene safety to rescuers takes precedence over patient safety. | 1C |
| 3 | Wear helmets to protect against traumatic brain injury (TBI). | 1B |
| 4 | Use additional safety equipment to prevent injuries and infection. | 1C |
| 5 | Consider a “grab and go” over a “stabilize on site” approach in a dangerous environment. | 1C |
|  | **A- Airway and C- Spine** |  |
| 6 | Provide oxygen (2B) especially at higher altitude (e.g. 2.500m a.s.l.) (1A). | 2B, 1A |
| 7 | Rescuers should be competent in opening, clearing and maintaining an airway patent. | 1C |
| 8 | Only experienced rescuers should perform prehospital tracheal intubation. | 1B |
| 9 | Consider advanced airway management with seriously impaired gas exchange. | 2B |
| 10 | Be cautious with advanced airway management prior to extrication by hoist because the artificial airway may dislocate or the patient being hypoventilated. | 1C |
| 11 | Consider a videolaryngoscope and an introducer to facilitate tracheal intubation. | 1B |
| 12 | Consider a supraglottic device as an alternative to tracheal intubation. | 2A |
| 13 | Reserve tracheal intubation for children to experienced rescuers and ventilate with bag-valve-mask instead. | 2B |
| 14 | After establishing an advanced airway use capnography to confirm correct position and guide normo-ventilation | 2B |
| 15 | Do not immobilize the C-spine of all blunt trauma patients nor neurologically intact patients with penetrating trauma. | 1A |
| 16 | Do not pre-hospitally clear the C-spine in children. | 1C |
| 17 | Immobilize the C-spine using manual in-line stabilization, SAM-splints or cervical collars. | 1B |
|  | **B - Breathing and Thoracic Injury** |  |
| 18 | Establish normoventilation with lung-protective ventilation and establish normoxia, and normocapnia in TBI patients. | 1A |
| 19 | Identify respiratory distress and use a pulse oximeter. | 1B |
| 20 | Consider the potential critical expansion of pneumothorax during helicopter evacuation. | 1B |
| 21 | If severe respiratory or circulatory compromise occur consider a deteriorating tension pneumothorax causative and immediately decompress the pleural cavity with a “mini-thoracostomy”  or pigtail catheter | 1B  2B |
|  | **C – Circulation and Bleeding Control** |  |
| 22 | Stop haemorrhage. | 1A |
| 23 | Maintain oxygenation and perfusion (mean arterial pressure (MAP) ≥65mmHg in priorly normotensive patients). | 1C |
| 24 | With uncontrolled haemorrhage, allow for permissive hypotension. | 1B |
| 25 | In case of severe haemorrhage rapid transport is key. | 1B |
| 26 | Bleeding control: First, attempt direct manual compression. | 1A |
| 27 | Use a modern tourniquet for uncontrolled extremity bleeding, it facilitates extraction. | 1B |
| 28 | Release tourniquet only after provision of definitive care.  Avoid release of the tourniquet every two hours. | 2B  2C |
| 29 | Consider non-compressible truncal haemorrhage control with expandable sponges and junctional tourniquets for the axilla and inguinal areas. | 2C |
| 30 | Consider temporary aortic occlusion with resuscitative balloon occlusion of the aorta (REBOA) for internal abdominopelvic haemorrhage. | 2C |
| 31 | Consider pelvic binders to close the pelvic ring. | 2C |
| 32 | Bleeding control: Administer TXA within three hours post-trauma. | 1B |
| 33 | Consider desmopressin for patients on platelet inhibitors, with von Willebrand disease. | 2A |
| 34 | Consider desmopressin for patients with hypothermia-induced hypocoagulation. | 2C |
| 35 | Consider fibrinogen concentrate administration over fresh frozen plasma (FFP). | 2B |
| 36 | Consider coagulation factor concentrates administration. | 2A |
| 37 | Consider reversal of coagulation factor inhibitors | 2A |
| 38 | Consider haemostatic dressings over plain gauze. | 1C |
| 39 | Establish at least one large bore IV-access for administration of fluid. | 1C |
| 40 | Establish IO access if IV access is impossible after three attempts and fluid and drug administration are required. | 1C |
| 41 | Consider rapid volume repletion judiciously to restore cardiac preload. | 1A |
| 42 | Consider norepinephrine in non-critically bleeding TBI patients to maintain cerebral perfusion pressure during prolonged transport. | 1C |
|  | **D- Disability and Neurotrauma** |  |
| 43 | In TBI, assess ventilation clinically and monitor patients with pulse-oximetry to minimise hypoxia. | 1C |
| 44 | Use capnography to establish normocapnia and established airway. | 1C |
| 45 | In TBI, aim for a systolic blood pressure ≥110 mmHg. | 2C |
| 46 | In TBI, expedite rescue and do not delay evacuation by maintaing head elevation | 1C |
| 47 | In TBI, administer tranexamic acid within the first three hours after trauma. | 1A |
| 48 | In TBI, avoid hypothermia. | 1C |
| 49 | Immobilise the spine of all multiple trauma patients when the conscious level is altered. | 1C |
| 50 | Use a clinical decision rule to identify patients at risk from secondary spinal injury and only if positive immobilize the spine. | 1B |
| 51 | Limit spinal motion with a combination of manual stabilisation, head blocks and hard or soft transfer devices. | 1C |
| 52 | For spine immobilisation, use techniques with minimal handling. | 1C |
| 53 | Do not log roll unstable trauma patients. | 1C |
| 54 | Consider a vacuum mattress for a long comfortable transfer. | 1C |
| 55 | Consider a KED or similar device for extrication in a non-horizontal position. | 1C |
|  | **E- Environment and Exposure** |  |
| 56 | For patient assessment, consider whether exposure is helpful in a given setting. | 1C |
| 57 | Exam sequentially by body regions avoiding heat loss and preserving insulating clothing. | 2C |
|  | **First aid, splinting and immobilisation** |  |
| 58 | Consider early splinting to reduce pain and blood loss and ease transportation. | 1C |
| 59 | Use splinting devices you are familiar with. | 1C |
| 60 | Consider the use of a vacuum splints. | 1C |
|  | **Analgesia** |  |
| 61 | Consider non-pharmacologic interventions (e.g. immobilization). | 1C |
| 62 | Consider the use of ketamine and other non-opioid analgesics (1C). Use opioids judiciously. | 1C |
| 63 | Consider regional anaesthesia in wilderness especially with painful injuries during long and difficult extrications or during fracture and joint reposition, repair and foreign body removal. | 1B |
| 64 | Regional anaesthesia may be ideal if respiratory compromise is of concern with systemic analgesia. | 1B |
| 65 | Ultrasound guidance of regional anaesthesia is recommended. | 1C |
|  | **Temperature Management and Hypothermia** |  |
| 66 | Without any diagnostic device use the Swiss staging system for hypothermia. | 1C |
| 67 | Epitympanic thermistor thermometers may be reliable for core temperature monitoring but lack of available equipment for field operations limit their use. | 1B |
| 68 | In patients with a secured airway consider core temperature measurement with an oesophageal probe. | 2A |
| 69 | A hypothermic patient should be extracted from the cold and covered by a tightly fitting vapor barrier followed by whole-body insulation. | 1C |
| 70 | Rewarming devices should be used in conjunction with vapor barriers and insulation. | 1C |
|  | **Transport and hospital selection** |  |
| 71 | Expedite transport of a multiple trauma patient. | 1C |
| 72 | Use light and stable stretchers for transport. | 1C |
| 73 | Avoid verticalizing the patient as this may lead to arterial hypotension especially in exsanguinated and vasoplegic patients. | 1C |
| 74 | Use helicopters for patients with spinal injury or who are haemodynamically unstable. | 1C |
| 75 | Transport TBI patients airborne if this saves time to definitive treatment. | 1B |
| 76 | Use helicopters to reach a distant high-level trauma centre, bypassing smaller local hospitals. | 1B |
| 77 | Balance the benefits of air rescue with its inherent risks. | 1C |
|  | **Ultrasound** |  |
| 78 | Consider point-of-care ultrasound (POCUS) in multiple trauma patients but do not considerably delay arrival to hospital. | 2B |
| 79 | Consider POCUS to manage a difficult airway. | 2C |
| 80 | Consider POCUS to detect pneumo- and haemothorax and pericardial effusion. | 1A |
| 81 | Consider POCUS to detect fractures. | 2B |
| 82 | Consider POCUS to detect increased intracerebral pressure. | 2B |
| 83 | Consider FAST in patients in shock. | 1B |
| 84 | Use ultrasound for vascular access and nerve blocks. | 1A |

**3. Literature**

1. Rauch S, Dal Cappello T, Strapazzon G, Palma M, Bonsante F, Gruber E, Strohle M, Mair P, Brugger H, International Alpine Trauma Registry Study G: **Pre-hospital times and clinical characteristics of severe trauma patients: A comparison between mountain and urban/suburban areas**. *Am J Emerg Med* 2018, **36**(10):1749-1753.

2. Hearns S: **The Scottish mountain rescue casualty study**. *Emerg Med J* 2003, **20**(3):281-284.

3. Gross T, Amsler F: **[Long-term outcome following multiple trauma in working age : A prospective study in a Swiss trauma center]**. *Unfallchirurg* 2016, **119**(11):921-928.

4. Gross T, Attenberger C, Huegli RW, Amsler F: **Factors associated with reduced longer-term capacity to work in patients after polytrauma: a Swiss trauma center experience**. *J Am Coll Surg* 2010, **211**(1):81-91.

5. Munn Z, Peters MDJ, Stern C, Tufanaru C, McArthur A, Aromataris E: **Systematic review or scoping review? Guidance for authors when choosing between a systematic or scoping review approach**. *BMC Med Res Methodol* 2018, **18**(1):143.

6. Guyatt G, Gutterman D, Baumann MH, Addrizzo-Harris D, Hylek EM, Phillips B, Raskob G, Lewis SZ, Schunemann H: **Grading strength of recommendations and quality of evidence in clinical guidelines: report from an american college of chest physicians task force**. *Chest* 2006, **129**(1):174-181.

7. Newgard CD, Schmicker RH, Hedges JR, Trickett JP, Davis DP, Bulger EM, Aufderheide TP, Minei JP, Hata JS, Gubler KD *et al*: **Emergency medical services intervals and survival in trauma: assessment of the "golden hour" in a North American prospective cohort**. *Ann Emerg Med* 2010, **55**(3):235-246 e234.

8. Newgard CD, Meier EN, Bulger EM, Buick J, Sheehan K, Lin S, Minei JP, Barnes-Mackey RA, Brasel K, Investigators ROC: **Revisiting the "Golden Hour": An Evaluation of Out-of-Hospital Time in Shock and Traumatic Brain Injury**. *Ann Emerg Med* 2015, **66**(1):30-41, 41 e31-33.

9. Ellerton J, Tomazin I, Brugger H, Paal P, International Commission for Mountain Emergency M: **Immobilization and splinting in mountain rescue. Official Recommendations of the International Commission for Mountain Emergency Medicine, ICAR MEDCOM, Intended for Mountain Rescue First Responders, Physicians, and Rescue Organizations**. *High Alt Med Biol* 2009, **10**(4):337-342.

10. **EMS, Tactical Care and Evacuation Under Fire** [https://www.ncbi.nlm.nih.gov/books/NBK441967/]

11. Baekgaard JS, Isbye D, Ottosen CI, Larsen MH, Andersen JH, Rasmussen LS, Steinmetz J: **Restrictive vs liberal oxygen for trauma patients-the TRAUMOX1 pilot randomised clinical trial**. *Acta Anaesthesiol Scand* 2019, **63**(7):947-955.

12. Lockey DJ, Healey B, Crewdson K, Chalk G, Weaver AE, Davies GE: **Advanced airway management is necessary in prehospital trauma patients**. *Br J Anaesth* 2015, **114**(4):657-662.

13. Pietsch U, Knapp J, Kreuzer O, Ney L, Strapazzon G, Lischke V, Albrecht R, Phillips P, Rauch S: **Advanced airway management in hoist and longline operations in mountain HEMS - considerations in austere environments: a narrative review This review is endorsed by the International Commission for Mountain Emergency Medicine (ICAR MEDCOM)**. *Scand J Trauma Resusc Emerg Med* 2018, **26**(1):23.

14. Angerman S, Kirves H, Nurmi J: **A before-and-after observational study of a protocol for use of the C-MAC videolaryngoscope with a Frova introducer in pre-hospital rapid sequence intubation**. *Anaesthesia* 2018, **73**(3):348-355.

15. Young B: **The intubating laryngeal-mask airway may be an ideal device for airway control in the rural trauma patient**. *Am J Emerg Med* 2003, **21**(1):80-85.

16. Tarpgaard M, Hansen TM, Rognas L: **Anaesthetist-provided pre-hospital advanced airway management in children: a descriptive study**. *Scand J Trauma Resusc Emerg Med* 2015, **23**:61.

17. Theodore N, Hadley MN, Aarabi B, Dhall SS, Gelb DE, Hurlbert RJ, Rozzelle CJ, Ryken TC, Walters BC: **Prehospital cervical spinal immobilization after trauma**. *Neurosurgery* 2013, **72 Suppl 2**:22-34.

18. Sundstrom T, Asbjornsen H, Habiba S, Sunde GA, Wester K: **Prehospital use of cervical collars in trauma patients: a critical review**. *J Neurotrauma* 2014, **31**(6):531-540.

19. Paterek E, Isenberg DL, Schiffer H: **Characteristics of Trauma Patients With Potential Cervical Spine Injuries Underimmobilized by Prehospital Providers**. *Spine (Phila Pa 1976)* 2015, **40**(24):1898-1902.

20. Velopulos CG, Shihab HM, Lottenberg L, Feinman M, Raja A, Salomone J, Haut ER: **Prehospital spine immobilization/spinal motion restriction in penetrating trauma: A practice management guideline from the Eastern Association for the Surgery of Trauma (EAST)**. *J Trauma Acute Care Surg* 2018, **84**(5):736-744.

21. Apfelbaum JD, Cantrill SV, Waldman N: **Unstable cervical spine without spinal cord injury in penetrating neck trauma**. *Am J Emerg Med* 2000, **18**(1):55-57.

22. Barkana Y, Stein M, Scope A, Maor R, Abramovich Y, Friedman Z, Knoller N: **Prehospital stabilization of the cervical spine for penetrating injuries of the neck - is it necessary?** *Injury* 2000, **31**(5):305-309.

23. Garcia A, Liu TH, Victorino GP: **Cost-utility analysis of prehospital spine immobilization recommendations for penetrating trauma**. *J Trauma Acute Care Surg* 2014, **76**(2):534-541.

24. Haut ER, Kalish BT, Efron DT, Haider AH, Stevens KA, Kieninger AN, Cornwell EE, 3rd, Chang DC: **Spine immobilization in penetrating trauma: more harm than good?** *J Trauma* 2010, **68**(1):115-120; discussion 120-111.

25. Klein Y, Arieli I, Sagiv S, Peleg K, Ben-Galim P: **Cervical spine injuries in civilian victims of explosions: Should cervical collars be used?** *J Trauma Acute Care Surg* 2016, **80**(6):985-988.

26. Hoffman JR, Wolfson AB, Todd K, Mower WR: **Selective cervical spine radiography in blunt trauma: methodology of the National Emergency X-Radiography Utilization Study (NEXUS)**. *Ann Emerg Med* 1998, **32**(4):461-469.

27. Hoffman JR, Mower WR, Wolfson AB, Todd KH, Zucker MI: **Validity of a set of clinical criteria to rule out injury to the cervical spine in patients with blunt trauma. National Emergency X-Radiography Utilization Study Group**. *N Engl J Med* 2000, **343**(2):94-99.

28. Domeier RM, Evans RW, Swor RA, Hancock JB, Fales W, Krohmer J, Frederiksen SM, Shork MA: **The reliability of prehospital clinical evaluation for potential spinal injury is not affected by the mechanism of injury**. *Prehosp Emerg Care* 1999, **3**(4):332-337.

29. Stroh G, Braude D: **Can an out-of-hospital cervical spine clearance protocol identify all patients with injuries? An argument for selective immobilization**. *Ann Emerg Med* 2001, **37**(6):609-615.

30. Vaillancourt C, Stiell IG, Beaudoin T, Maloney J, Anton AR, Bradford P, Cain E, Travers A, Stempien M, Lees M *et al*: **The out-of-hospital validation of the Canadian C-Spine Rule by paramedics**. *Ann Emerg Med* 2009, **54**(5):663-671 e661.

31. Slaar A, Fockens MM, Wang J, Maas M, Wilson DJ, Goslings JC, Schep NW, van Rijn RR: **Triage tools for detecting cervical spine injury in pediatric trauma patients**. *Cochrane Database Syst Rev* 2017, **12**:CD011686.

32. Lee SL, Sena M, Greenholz SK, Fledderman M: **A multidisciplinary approach to the development of a cervical spine clearance protocol: process, rationale, and initial results**. *J Pediatr Surg* 2003, **38**(3):358-362; discussion 358-362.

33. Chan M, Al-Buali W, Charyk Stewart T, Singh RN, Kornecki A, Seabrook JA, Fraser DD: **Cervical spine injuries and collar complications in severely injured paediatric trauma patients**. *Spinal Cord* 2013, **51**(5):360-364.

34. Viccellio P, Simon H, Pressman BD, Shah MN, Mower WR, Hoffman JR, Group N: **A prospective multicenter study of cervical spine injury in children**. *Pediatrics* 2001, **108**(2):E20.

35. McGrath T, Murphy C: **Comparison of a SAM splint-molded cervical collar with a Philadelphia cervical collar**. *Wilderness Environ Med* 2009, **20**(2):166-168.

36. Kreinest M, Goller S, Rauch G, Frank C, Gliwitzky B, Wolfl CG, Matschke S, Munzberg M: **Application of Cervical Collars - An Analysis of Practical Skills of Professional Emergency Medical Care Providers**. *PLoS One* 2015, **10**(11):e0143409.

37. Davies G, Deakin C, Wilson A: **The effect of a rigid collar on intracranial pressure**. *Injury* 1996, **27**(9):647-649.

38. Kolb JC, Summers RL, Galli RL: **Cervical collar-induced changes in intracranial pressure**. *Am J Emerg Med* 1999, **17**(2):135-137.

39. Karason S, Reynisson K, Sigvaldason K, Sigurdsson GH: **Evaluation of clinical efficacy and safety of cervical trauma collars: differences in immobilization, effect on jugular venous pressure and patient comfort**. *Scand J Trauma Resusc Emerg Med* 2014, **22**:37.

40. Stone MB, Tubridy CM, Curran R: **The effect of rigid cervical collars on internal jugular vein dimensions**. *Acad Emerg Med* 2010, **17**(1):100-102.

41. Woster CM, Zwank MD, Pasquarella JR, Wewerka SS, Anderson JP, Greupner JT, Motalib S: **Placement of a cervical collar increases the optic nerve sheath diameter in healthy adults**. *Am J Emerg Med* 2018, **36**(3):430-434.

42. Helm M, Schuster R, Hauke J, Lampl L: **Tight control of prehospital ventilation by capnography in major trauma victims**. *Br J Anaesth* 2003, **90**(3):327-332.

43. Haider T, Halat G, Heinz T, Hajdu S, Negrin LL: **Thoracic trauma and acute respiratory distress syndrome in polytraumatized patients: a retrospective analysis**. *Minerva Anestesiol* 2017, **83**(10):1026-1033.

44. Helm M, Hauke J, Esser M, Lampl L, Bock KH: **[Diagnosis of blunt thoracic trauma in emergency care. Use of continuous pulse oximetry monitoring]**. *Chirurg* 1997, **68**(6):606-612.

45. Knotts D, Arthur AO, Holder P, Herrington T, Thomas SH: **Pneumothorax volume expansion in helicopter emergency medical services transport**. *Air Med J* 2013, **32**(3):138-143.

46. Leigh-Smith S, Harris T: **Tension pneumothorax--time for a re-think?** *Emerg Med J* 2005, **22**(1):8-16.

47. Lesperance RN, Carroll CM, Aden JK, Young JB, Nunez TC: **Failure Rate of Prehospital Needle Decompression for Tension Pneumothorax in Trauma Patients**. *Am Surg* 2018, **84**(11):1750-1755.

48. Chan L, Reilly KM, Henderson C, Kahn F, Salluzzo RF: **Complication rates of tube thoracostomy**. *Am J Emerg Med* 1997, **15**(4):368-370.

49. Fang M, Liu G, Luo G, Wu T: **Does pigtail catheters relieve pneumothorax?: A PRISMA-compliant systematic review and meta-analysis**. *Medicine (Baltimore)* 2018, **97**(47):e13255.

50. Bauman ZM, Kulvatunyou N, Joseph B, Jain A, Friese RS, Gries L, O'Keeffe T, Tang AL, Vercruysse G, Rhee P: **A Prospective Study of 7-Year Experience Using Percutaneous 14-French Pigtail Catheters for Traumatic Hemothorax/Hemopneumothorax at a Level-1 Trauma Center: Size Still Does Not Matter**. *World J Surg* 2018, **42**(1):107-113.

51. Hyde J, Sykes T, Graham T: **Reducing morbidity from chest drains**. *BMJ* 1997, **314**(7085):914-915.

52. Valdez C, Sarani B, Young H, Amdur R, Dunne J, Chawla LS: **Timing of death after traumatic injury--a contemporary assessment of the temporal distribution of death**. *J Surg Res* 2016, **200**(2):604-609.

53. Kohli-Seth R, Neuman T, Sinha R, Bassily-Marcus A: **Use of echocardiography and modalities of patient monitoring of trauma patients**. *Curr Opin Anaesthesiol* 2010, **23**(2):239-245.

54. Rossaint R, Bouillon B, Cerny V, Coats TJ, Duranteau J, Fernandez-Mondejar E, Filipescu D, Hunt BJ, Komadina R, Nardi G *et al*: **The European guideline on management of major bleeding and coagulopathy following trauma: fourth edition**. *Crit Care* 2016, **20**:100.

55. Bouillon B, Marzi I: **The updated German "Polytrauma - Guideline": an extensive literature evaluation and treatment recommendation for the care of the critically injured patient**. *Eur J Trauma Emerg Surg* 2018, **44**(Suppl 1):1.

56. Bulger EM, Snyder D, Schoelles K, Gotschall C, Dawson D, Lang E, Sanddal ND, Butler FK, Fallat M, Taillac P *et al*: **An evidence-based prehospital guideline for external hemorrhage control: American College of Surgeons Committee on Trauma**. *Prehosp Emerg Care* 2014, **18**(2):163-173.

57. Singletary EM, Charlton NP, Epstein JL, Ferguson JD, Jensen JL, MacPherson AI, Pellegrino JL, Smith WW, Swain JM, Lojero-Wheatley LF *et al*: **Part 15: First Aid: 2015 American Heart Association and American Red Cross Guidelines Update for First Aid**. *Circulation* 2015, **132**(18 Suppl 2):S574-589.

58. Spahn DR, Bouillon B, Cerny V, Duranteau J, Filipescu D, Hunt BJ, Komadina R, Maegele M, Nardi G, Riddez L *et al*: **The European guideline on management of major bleeding and coagulopathy following trauma: fifth edition**. *Crit Care* 2019, **23**(1):98.

59. Chang R, Eastridge BJ, Holcomb JB: **Remote Damage Control Resuscitation in Austere Environments**. *Wilderness Environ Med* 2017, **28**(2S):S124-S134.

60. Kragh JF, Jr., Dubick MA: **Bleeding Control With Limb Tourniquet Use in the Wilderness Setting: Review of Science**. *Wilderness Environ Med* 2017, **28**(2S):S25-S32.

61. Scerbo MH, Holcomb JB, Taub E, Gates K, Love JD, Wade CE, Cotton BA: **The trauma center is too late: Major limb trauma without a pre-hospital tourniquet has increased death from hemorrhagic shock**. *J Trauma Acute Care Surg* 2017, **83**(6):1165-1172.

62. Inaba K, Siboni S, Resnick S, Zhu J, Wong MD, Haltmeier T, Benjamin E, Demetriades D: **Tourniquet use for civilian extremity trauma**. *J Trauma Acute Care Surg* 2015, **79**(2):232-237;quiz 332-233.

63. Dayan L, Zinmann C, Stahl S, Norman D: **Complications associated with prolonged tourniquet application on the battlefield**. *Mil Med* 2008, **173**(1):63-66.

64. Warriner Z, Lam L, Matsushima K, Benjamin E, Strumwasser A, Demetriades D, Inaba K: **Initial evaluation of the efficacy and safety of in-hospital expandable hemostatic minisponge use in penetrating trauma**. *J Trauma Acute Care Surg* 2019, **86**(3):424-430.

65. Smith S, White J, Wanis KN, Beckett A, McAlister VC, Hilsden R: **The effectiveness of junctional tourniquets: A systematic review and meta-analysis**. *J Trauma Acute Care Surg* 2019, **86**(3):532-539.

66. Borger van der Burg BLS, van Dongen T, Morrison JJ, Hedeman Joosten PPA, DuBose JJ, Horer TM, Hoencamp R: **A systematic review and meta-analysis of the use of resuscitative endovascular balloon occlusion of the aorta in the management of major exsanguination**. *Eur J Trauma Emerg Surg* 2018, **44**(4):535-550.

67. Davidson AJ, Russo RM, Reva VA, Brenner ML, Moore LJ, Ball C, Bulger E, Fox CJ, DuBose JJ, Moore EE *et al*: **The pitfalls of resuscitative endovascular balloon occlusion of the aorta: Risk factors and mitigation strategies**. *J Trauma Acute Care Surg* 2018, **84**(1):192-202.

68. Vermeulen B, Peter R, Hoffmeyer P, Unger PF: **Prehospital stabilization of pelvic dislocations: a new strap belt to provide temporary hemodynamic stabilization**. *Swiss Surg* 1999, **5**(2):43-46.

69. Qureshi A, McGee A, Cooper JP, Porter KM: **Reduction of the posterior pelvic ring by non-invasive stabilisation: a report of two cases**. *Emerg Med J* 2005, **22**(12):885-886.

70. Bakhshayesh P, Boutefnouchet T, Totterman A: **Effectiveness of non invasive external pelvic compression: a systematic review of the literature**. *Scand J Trauma Resusc Emerg Med* 2016, **24**:73.

71. Hoch A, Zeidler S, Pieroh P, Josten C, Stuby FM, Herath SC, German Pelvic Trauma R: **Trends and efficacy of external emergency stabilization of pelvic ring fractures: results from the German Pelvic Trauma Registry**. *Eur J Trauma Emerg Surg* 2019.

72. collaborators C-t, Shakur H, Roberts I, Bautista R, Caballero J, Coats T, Dewan Y, El-Sayed H, Gogichaishvili T, Gupta S *et al*: **Effects of tranexamic acid on death, vascular occlusive events, and blood transfusion in trauma patients with significant haemorrhage (CRASH-2): a randomised, placebo-controlled trial**. *Lancet* 2010, **376**(9734):23-32.

73. Roberts I, Shakur H, Ker K, Coats T, collaborators C-T: **Antifibrinolytic drugs for acute traumatic injury**. *Cochrane Database Syst Rev* 2012, **12**:CD004896.

74. Roberts I, Prieto-Merino D, Manno D: **Mechanism of action of tranexamic acid in bleeding trauma patients: an exploratory analysis of data from the CRASH-2 trial**. *Crit Care* 2014, **18**(6):685.

75. Roberts I, Coats T, Edwards P, Gilmore I, Jairath V, Ker K, Manno D, Shakur H, Stanworth S, Veitch A: **HALT-IT--tranexamic acid for the treatment of gastrointestinal bleeding: study protocol for a randomised controlled trial**. *Trials* 2014, **15**:450.

76. Huebner BR, Dorlac WC, Cribari C: **Tranexamic Acid Use in Prehospital Uncontrolled Hemorrhage**. *Wilderness Environ Med* 2017, **28**(2S):S50-S60.

77. Wafaisade A, Lefering R, Bouillon B, Bohmer AB, Gassler M, Ruppert M, TraumaRegister DGU: **Prehospital administration of tranexamic acid in trauma patients**. *Crit Care* 2016, **20**(1):143.

78. Cole E, Davenport R, Willett K, Brohi K: **Tranexamic acid use in severely injured civilian patients and the effects on outcomes: a prospective cohort study**. *Ann Surg* 2015, **261**(2):390-394.

79. Reiter RA, Mayr F, Blazicek H, Galehr E, Jilma-Stohlawetz P, Domanovits H, Jilma B: **Desmopressin antagonizes the in vitro platelet dysfunction induced by GPIIb/IIIa inhibitors and aspirin**. *Blood* 2003, **102**(13):4594-4599.

80. Leithauser B, Zielske D, Seyfert UT, Jung F: **Effects of desmopressin on platelet membrane glycoproteins and platelet aggregation in volunteers on clopidogrel**. *Clin Hemorheol Microcirc* 2008, **39**(1-4):293-302.

81. Laupacis A, Fergusson D: **Drugs to minimize perioperative blood loss in cardiac surgery: meta-analyses using perioperative blood transfusion as the outcome. The International Study of Peri-operative Transfusion (ISPOT) Investigators**. *Anesth Analg* 1997, **85**(6):1258-1267.

82. McMillian WD, Rogers FB: **Management of prehospital antiplatelet and anticoagulant therapy in traumatic head injury: a review**. *J Trauma* 2009, **66**(3):942-950.

83. Powner DJ, Hartwell EA, Hoots WK: **Counteracting the effects of anticoagulants and antiplatelet agents during neurosurgical emergencies**. *Neurosurgery* 2005, **57**(5):823-831; discussion 823-831.

84. Kapapa T, Rohrer S, Struve S, Petscher M, Konig R, Wirtz CR, Woischneck D: **Desmopressin acetate in intracranial haemorrhage**. *Neurol Res Int* 2014, **2014**:298767.

85. Levine M, Swenson S, McCormick T, Henderson SO, Thomas SH, Markland FS: **Reversal of thienopyridine-induced platelet dysfunction following desmopressin administration**. *J Med Toxicol* 2013, **9**(2):139-143.

86. Teng R, Mitchell PD, Butler K: **The effect of desmopressin on bleeding time and platelet aggregation in healthy volunteers administered ticagrelor**. *J Clin Pharm Ther* 2014, **39**(2):186-191.

87. Castaman G, Linari S: **Diagnosis and Treatment of von Willebrand Disease and Rare Bleeding Disorders**. *J Clin Med* 2017, **6**(4).

88. Coppola A, Di Minno G: **Desmopressin in inherited disorders of platelet function**. *Haemophilia* 2008, **14 Suppl 1**:31-39.

89. Ng KF, Cheung CW, Lee Y, Leung SW: **Low-dose desmopressin improves hypothermia-induced impairment of primary haemostasis in healthy volunteers**. *Anaesthesia* 2011, **66**(11):999-1005.

90. Hanke AA, Dellweg C, Kienbaum P, Weber CF, Gorlinger K, Rahe-Meyer N: **Effects of desmopressin on platelet function under conditions of hypothermia and acidosis: an in vitro study using multiple electrode aggregometry***. *Anaesthesia* 2010, **65**(7):688-691.

91. Crescenzi G, Landoni G, Biondi-Zoccai G, Pappalardo F, Nuzzi M, Bignami E, Fochi O, Maj G, Calabro MG, Ranucci M *et al*: **Desmopressin reduces transfusion needs after surgery: a meta-analysis of randomized clinical trials**. *Anesthesiology* 2008, **109**(6):1063-1076.

92. Yamamoto K, Yamaguchi A, Sawano M, Matsuda M, Anan M, Inokuchi K, Sugiyama S: **Pre-emptive administration of fibrinogen concentrate contributes to improved prognosis in patients with severe trauma**. *Trauma Surg Acute Care Open* 2016, **1**(1):e000037.

93. Innerhofer P, Fries D, Mittermayr M, Innerhofer N, von Langen D, Hell T, Gruber G, Schmid S, Friesenecker B, Lorenz IH *et al*: **Reversal of trauma-induced coagulopathy using first-line coagulation factor concentrates or fresh frozen plasma (RETIC): a single-centre, parallel-group, open-label, randomised trial**. *Lancet Haematol* 2017, **4**(6):e258-e271.

94. Matsushima K, Benjamin E, Demetriades D: **Prothrombin complex concentrate in trauma patients**. *Am J Surg* 2015, **209**(2):413-417.

95. Berger K, Santibanez M, Lin L, Lesch CA: **A Low-Dose 4F-PCC Protocol for DOAC-Associated Intracranial Hemorrhage**. *J Intensive Care Med* 2019:885066619840992.

96. Sarode R, Milling TJ, Jr., Refaai MA, Mangione A, Schneider A, Durn BL, Goldstein JN: **Efficacy and safety of a 4-factor prothrombin complex concentrate in patients on vitamin K antagonists presenting with major bleeding: a randomized, plasma-controlled, phase IIIb study**. *Circulation* 2013, **128**(11):1234-1243.

97. Goldstein JN, Refaai MA, Milling TJ, Jr., Lewis B, Goldberg-Alberts R, Hug BA, Sarode R: **Four-factor prothrombin complex concentrate versus plasma for rapid vitamin K antagonist reversal in patients needing urgent surgical or invasive interventions: a phase 3b, open-label, non-inferiority, randomised trial**. *Lancet* 2015, **385**(9982):2077-2087.

98. Bennett BL: **Bleeding Control Using Hemostatic Dressings: Lessons Learned**. *Wilderness Environ Med* 2017, **28**(2S):S39-S49.

99. Kozen BG, Kircher SJ, Henao J, Godinez FS, Johnson AS: **An alternative hemostatic dressing: comparison of CELOX, HemCon, and QuikClot**. *Acad Emerg Med* 2008, **15**(1):74-81.

100. Allison HA: **Hemorrhage Control: Lessons Learned From the Battlefield Use of Hemostatic Agents That Can Be Applied in a Hospital Setting**. *Crit Care Nurs Q* 2019, **42**(2):165-172.

101. Peters JH, Smulders PSH, Moors XRJ, Bouman SJM, Meijs C, Hoogerwerf N, Edwards MJR: **Are on-scene blood transfusions by a helicopter emergency medical service useful and safe? A multicentre case-control study**. *Eur J Emerg Med* 2019, **26**(2):128-132.

102. Sato Folatre JG, Arnell P, Henning M, Josefsson K, Skallsjo G, Ricksten SE: **[Introduction of prehospital blood transfusion programme in Sweden: experiences from a physician staffed helicopter emergency medical service]**. *Lakartidningen* 2018, **115**.

103. Krook C, O'Dochartaigh D, Martin D, Piggott Z, Deedo R, Painter S, van Werkhoven G, McKay D, Nesdoly D, Armstrong JN: **Blood on board: The development of a prehospital blood transfusion program in a Canadian helicopter emergency medical service**. *CJEM* 2019, **21**(3):365-373.

104. Moors XRJ, Bouman SJM, Peters JH, Smulders P, Alink MBO, Hartog DD, Stolker RJ: **Prehospital Blood Transfusions in Pediatric Patients by a Helicopter Emergency Medical Service**. *Air Med J* 2018, **37**(5):321-324.

105. Gurney JM, Spinella PC: **Blood transfusion management in the severely bleeding military patient**. *Curr Opin Anaesthesiol* 2018, **31**(2):207-214.

106. Semler MW, Self WH, Wanderer JP, Ehrenfeld JM, Wang L, Byrne DW, Stollings JL, Kumar AB, Hughes CG, Hernandez A *et al*: **Balanced Crystalloids versus Saline in Critically Ill Adults**. *N Engl J Med* 2018, **378**(9):829-839.

107. Sarrafzadeh AS, Peltonen EE, Kaisers U, Kuchler I, Lanksch WR, Unterberg AW: **Secondary insults in severe head injury--do multiply injured patients do worse?** *Crit Care Med* 2001, **29**(6):1116-1123.

108. Chesnut RM, Marshall LF, Klauber MR, Blunt BA, Baldwin N, Eisenberg HM, Jane JA, Marmarou A, Foulkes MA: **The role of secondary brain injury in determining outcome from severe head injury**. *J Trauma* 1993, **34**(2):216-222.

109. Karamanos E, Talving P, Skiada D, Osby M, Inaba K, Lam L, Albuz O, Demetriades D: **Is prehospital endotracheal intubation associated with improved outcomes in isolated severe head injury? A matched cohort analysis**. *Prehosp Disaster Med* 2014, **29**(1):32-36.

110. Bossers SM, Schwarte LA, Loer SA, Twisk JW, Boer C, Schober P: **Experience in Prehospital Endotracheal Intubation Significantly Influences Mortality of Patients with Severe Traumatic Brain Injury: A Systematic Review and Meta-Analysis**. *PLoS One* 2015, **10**(10):e0141034.

111. Haltmeier T, Benjamin E, Siboni S, Dilektasli E, Inaba K, Demetriades D: **Prehospital intubation for isolated severe blunt traumatic brain injury: worse outcomes and higher mortality**. *Eur J Trauma Emerg Surg* 2017, **43**(6):731-739.

112. Davis DP, Peay J, Sise MJ, Vilke GM, Kennedy F, Eastman AB, Velky T, Hoyt DB: **The impact of prehospital endotracheal intubation on outcome in moderate to severe traumatic brain injury**. *J Trauma* 2005, **58**(5):933-939.

113. Wang HE, Brown SP, MacDonald RD, Dowling SK, Lin S, Davis D, Schreiber MA, Powell J, van Heest R, Daya M: **Association of out-of-hospital advanced airway management with outcomes after traumatic brain injury and hemorrhagic shock in the ROC hypertonic saline trial**. *Emerg Med J* 2014, **31**(3):186-191.

114. Bulger EM, Copass MK, Sabath DR, Maier RV, Jurkovich GJ: **The use of neuromuscular blocking agents to facilitate prehospital intubation does not impair outcome after traumatic brain injury**. *J Trauma* 2005, **58**(4):718-723; discussion 723-714.

115. Seo DE, Shin SD, Song KJ, Ro YS, Hong KJ, Park JH: **Effect of hypoxia on mortality and disability in traumatic brain injury according to shock status: A cross-sectional analysis**. *Am J Emerg Med* 2019, **37**(9):1709-1715.

116. Warner KJ, Cuschieri J, Copass MK, Jurkovich GJ, Bulger EM: **The impact of prehospital ventilation on outcome after severe traumatic brain injury**. *J Trauma* 2007, **62**(6):1330-1336; discussion 1336-1338.

117. Helm M, Hauke J, Lampl L: **A prospective study of the quality of pre-hospital emergency ventilation in patients with severe head injury**. *Br J Anaesth* 2002, **88**(3):345-349.

118. Hill DA, Abraham KJ, West RH: **Factors affecting outcome in the resuscitation of severely injured patients**. *Aust N Z J Surg* 1993, **63**(8):604-609.

119. Butcher I, Maas AI, Lu J, Marmarou A, Murray GD, Mushkudiani NA, McHugh GS, Steyerberg EW: **Prognostic value of admission blood pressure in traumatic brain injury: results from the IMPACT study**. *J Neurotrauma* 2007, **24**(2):294-302.

120. Berry C, Ley EJ, Bukur M, Malinoski D, Margulies DR, Mirocha J, Salim A: **Redefining hypotension in traumatic brain injury**. *Injury* 2012, **43**(11):1833-1837.

121. Spaite DW, Hu C, Bobrow BJ, Chikani V, Barnhart B, Gaither JB, Denninghoff KR, Adelson PD, Keim SM, Viscusi C *et al*: **Association of Out-of-Hospital Hypotension Depth and Duration With Traumatic Brain Injury Mortality**. *Ann Emerg Med* 2017, **70**(4):522-530 e521.

122. Eastridge BJ, Salinas J, McManus JG, Blackburn L, Bugler EM, Cooke WH, Convertino VA, Wade CE, Holcomb JB: **Hypotension begins at 110 mm Hg: redefining "hypotension" with data**. *J Trauma* 2007, **63**(2):291-297; discussion 297-299.

123. Carney N, Totten AM, O'Reilly C, Ullman JS, Hawryluk GW, Bell MJ, Bratton SL, Chesnut R, Harris OA, Kissoon N *et al*: **Guidelines for the Management of Severe Traumatic Brain Injury, Fourth Edition**. *Neurosurgery* 2017, **80**(1):6-15.

124. Brenner M, Stein DM, Hu PF, Aarabi B, Sheth K, Scalea TM: **Traditional systolic blood pressure targets underestimate hypotension-induced secondary brain injury**. *J Trauma Acute Care Surg* 2012, **72**(5):1135-1139.

125. Kannan N, Wang J, Mink RB, Wainwright MS, Groner JI, Bell MJ, Giza CC, Zatzick DF, Ellenbogen RG, Boyle LN *et al*: **Timely Hemodynamic Resuscitation and Outcomes in Severe Pediatric Traumatic Brain Injury: Preliminary Findings**. *Pediatr Emerg Care* 2018, **34**(5):325-329.

126. Cooper DJ, Myles PS, McDermott FT, Murray LJ, Laidlaw J, Cooper G, Tremayne AB, Bernard SS, Ponsford J, Investigators HTSS: **Prehospital hypertonic saline resuscitation of patients with hypotension and severe traumatic brain injury: a randomized controlled trial**. *JAMA* 2004, **291**(11):1350-1357.

127. Sumann G, Paal P, Mair P, Ellerton J, Dahlberg T, Zen-Ruffinen G, Zafren K, Brugger H: **Fluid management in traumatic shock: a practical approach for mountain rescue. Official recommendations of the International Commission for Mountain Emergency Medicine (ICAR MEDCOM)**. *High Alt Med Biol* 2009, **10**(1):71-75.

128. Hylands M, Toma A, Beaudoin N, Frenette AJ, D'Aragon F, Belley-Cote E, Charbonney E, Moller MH, Laake JH, Vandvik PO *et al*: **Early vasopressor use following traumatic injury: a systematic review**. *BMJ Open* 2017, **7**(11):e017559.

129. Rickard AC, Smith JE, Newell P, Bailey A, Kehoe A, Mann C: **Salt or sugar for your injured brain? A meta-analysis of randomised controlled trials of mannitol versus hypertonic sodium solutions to manage raised intracranial pressure in traumatic brain injury**. *Emerg Med J* 2014, **31**(8):679-683.

130. Sakellaridis N, Pavlou E, Karatzas S, Chroni D, Vlachos K, Chatzopoulos K, Dimopoulou E, Kelesis C, Karaouli V: **Comparison of mannitol and hypertonic saline in the treatment of severe brain injuries**. *J Neurosurg* 2011, **114**(2):545-548.

131. Wakai A, Roberts I, Schierhout G: **Mannitol for acute traumatic brain injury**. *Cochrane Database Syst Rev* 2007(1):CD001049.

132. Wang K, Sun M, Jiang H, Cao XP, Zeng J: **Mannitol cannot reduce the mortality on acute severe traumatic brain injury (TBI) patients: a meta-analyses and systematic review**. *Burns Trauma* 2015, **3**:8.

133. Agbeko RS, Pearson S, Peters MJ, McNames J, Goldstein B: **Intracranial pressure and cerebral perfusion pressure responses to head elevation changes in pediatric traumatic brain injury**. *Pediatr Crit Care Med* 2012, **13**(1):e39-47.

134. Alarcon JD, Rubiano AM, Okonkwo DO, Alarcon J, Martinez-Zapata MJ, Urrutia G, Bonfill Cosp X: **Elevation of the head during intensive care management in people with severe traumatic brain injury**. *Cochrane Database Syst Rev* 2017, **12**:CD009986.

135. collaborators C-t: **Effects of tranexamic acid on death, disability, vascular occlusive events and other morbidities in patients with acute traumatic brain injury (CRASH-3): a randomised, placebo-controlled trial**. *Lancet* 2019, **394**(10210):1713-1723.

136. Weng S, Wang W, Wei Q, Lan H, Su J, Xu Y: **Effect of Tranexamic Acid in Patients with Traumatic Brain Injury: A Systematic Review and Meta-Analysis**. *World Neurosurg* 2019, **123**:128-135.

137. Harris T, Davenport R, Hurst T, Jones J: **Improving outcome in severe trauma: trauma systems and initial management: intubation, ventilation and resuscitation**. *Postgrad Med J* 2012, **88**(1044):588-594.

138. Jeremitsky E, Omert L, Dunham CM, Protetch J, Rodriguez A: **Harbingers of poor outcome the day after severe brain injury: hypothermia, hypoxia, and hypoperfusion**. *J Trauma* 2003, **54**(2):312-319.

139. Lewis SR, Evans DJ, Butler AR, Schofield-Robinson OJ, Alderson P: **Hypothermia for traumatic brain injury**. *Cochrane Database Syst Rev* 2017, **9**:CD001048.

140. Cooper DJ, Nichol AD, Bailey M, Bernard S, Cameron PA, Pili-Floury S, Forbes A, Gantner D, Higgins AM, Huet O *et al*: **Effect of Early Sustained Prophylactic Hypothermia on Neurologic Outcomes Among Patients With Severe Traumatic Brain Injury: The POLAR Randomized Clinical Trial**. *JAMA* 2018, **320**(21):2211-2220.

141. Clifton GL, Valadka A, Zygun D, Coffey CS, Drever P, Fourwinds S, Janis LS, Wilde E, Taylor P, Harshman K *et al*: **Very early hypothermia induction in patients with severe brain injury (the National Acute Brain Injury Study: Hypothermia II): a randomised trial**. *Lancet Neurol* 2011, **10**(2):131-139.

142. Kwan I, Bunn F, Roberts I: **Spinal immobilisation for trauma patients**. *Cochrane Database Syst Rev* 2001(2):CD002803.

143. Hauswald M, Ong G, Tandberg D, Omar Z: **Out-of-hospital spinal immobilization: its effect on neurologic injury**. *Acad Emerg Med* 1998, **5**(3):214-219.

144. Hyldmo PK, Vist GE, Feyling AC, Rognas L, Magnusson V, Sandberg M, Soreide E: **Is the supine position associated with loss of airway patency in unconscious trauma patients? A systematic review and meta-analysis**. *Scand J Trauma Resusc Emerg Med* 2015, **23**:50.

145. Kwan I, Bunn F: **Effects of prehospital spinal immobilization: a systematic review of randomized trials on healthy subjects**. *Prehosp Disaster Med* 2005, **20**(1):47-53.

146. Quinn R, Williams J, Bennett B, Stiller G, Islas A, McCord S, Wilderness Medical S: **Wilderness Medical Society practice guidelines for spine immobilization in the austere environment**. *Wilderness Environ Med* 2013, **24**(3):241-252.

147. Kornhall DK, Jorgensen JJ, Brommeland T, Hyldmo PK, Asbjornsen H, Dolven T, Hansen T, Jeppesen E: **The Norwegian guidelines for the prehospital management of adult trauma patients with potential spinal injury**. *Scand J Trauma Resusc Emerg Med* 2017, **25**(1):2.

148. Todd NV, Skinner D, Wilson-MacDonald J: **Secondary neurological deterioration in traumatic spinal injury: data from medicolegal cases**. *Bone Joint J* 2015, **97-B**(4):527-531.

149. Boissy P, Shrier I, Briere S, Mellete J, Fecteau L, Matheson GO, Garza D, Meeuwisse WH, Segal E, Boulay J *et al*: **Effectiveness of cervical spine stabilization techniques**. *Clin J Sport Med* 2011, **21**(2):80-88.

150. Raphael JH, Chotai R: **Effects of the cervical collar on cerebrospinal fluid pressure**. *Anaesthesia* 1994, **49**(5):437-439.

151. Bruijns SR, Guly HR, Wallis LA: **Effect of spinal immobilization on heart rate, blood pressure and respiratory rate**. *Prehosp Disaster Med* 2013, **28**(3):210-214.

152. Holla M: **Value of a rigid collar in addition to head blocks: a proof of principle study**. *Emerg Med J* 2012, **29**(2):104-107.

153. Conrad BP, Horodyski M, Wright J, Ruetz P, Rechtine GR, 2nd: **Log-rolling technique producing unacceptable motion during body position changes in patients with traumatic spinal cord injury**. *J Neurosurg Spine* 2007, **6**(6):540-543.

154. Mahshidfar B, Mofidi M, Yari AR, Mehrsorosh S: **Long backboard versus vacuum mattress splint to immobilize whole spine in trauma victims in the field: a randomized clinical trial**. *Prehosp Disaster Med* 2013, **28**(5):462-465.

155. Luscombe MD, Williams JL: **Comparison of a long spinal board and vacuum mattress for spinal immobilisation**. *Emerg Med J* 2003, **20**(5):476-478.

156. Hamilton RS, Pons PT: **The efficacy and comfort of full-body vacuum splints for cervical-spine immobilization**. *J Emerg Med* 1996, **14**(5):553-559.

157. McDonald N, Webster M, Orkin A, VanderBurgh D, Johnson DE: **The Long Backboard vs the Vacuum Mattress**. *Prehosp Disaster Med* 2014, **29**(1):110.

158. Winterberger E, Jacomet H, Zafren K, Ruffinen GZ, Jelk B, International Commission for Mountain Emergency M, Terrestrial Rescue Commission of the International Commission for Alpine R: **The use of extrication devices in crevasse accidents: official statement of the International Commission for Mountain Emergency Medicine and the Terrestrial Rescue Commission of the International Commission for Alpine Rescue intended for physicians, paramedics, and mountain rescuers**. *Wilderness Environ Med* 2008, **19**(2):108-110.

159. Vayer JS, Hagmann JH, Llewellyn CH: **Refining prehospital physical assessment skills: a new teaching technique**. *Ann Emerg Med* 1994, **23**(4):786-790.

160. Butler FK, Bennett B, Wedmore CI: **Tactical Combat Casualty Care and Wilderness Medicine: Advancing Trauma Care in Austere Environments**. *Emerg Med Clin North Am* 2017, **35**(2):391-407.

161. Wolberg AS, Meng ZH, Monroe DM, 3rd, Hoffman M: **A systematic evaluation of the effect of temperature on coagulation enzyme activity and platelet function**. *J Trauma* 2004, **56**(6):1221-1228.

162. Beal AL, Ahrendt MN, Irwin ED, Lyng JW, Turner SV, Beal CA, Byrnes MT, Beilman GA: **Prediction of blunt traumatic injuries and hospital admission based on history and physical exam**. *World J Emerg Surg* 2016, **11**(1):46.

163. Esmer E, Derst P, Lefering R, Schulz M, Siekmann H, Delank KS, das TraumaRegister DGU: **[Prehospital assessment of injury type and severity in severely injured patients by emergency physicians : An analysis of the TraumaRegister DGU(R)]**. *Unfallchirurg* 2017, **120**(5):409-416.

164. McIntosh SE, Leemon D, Visitacion J, Schimelpfenig T, Fosnocht D: **Medical incidents and evacuations on wilderness expeditions**. *Wilderness Environ Med* 2007, **18**(4):298-304.

165. Lee C, Porter KM: **Prehospital management of lower limb fractures**. *Emerg Med J* 2005, **22**(9):660-663.

166. Agrawal Y, Karwa J, Shah N, Clayson A: **Traction splint: to use or not to use**. *J Perioper Pract* 2009, **19**(9):295-298.

167. Wood SP, Vrahas M, Wedel SK: **Femur fracture immobilization with traction splints in multisystem trauma patients**. *Prehosp Emerg Care* 2003, **7**(2):241-243.

168. Abarbanell NR: **Prehospital midthigh trauma and traction splint use: recommendations for treatment protocols**. *Am J Emerg Med* 2001, **19**(2):137-140.

169. Quinn RH, Macias DJ: **The management of open fractures**. *Wilderness Environ Med* 2006, **17**(1):41-48.

170. Rowlands TK, Clasper J: **The Thomas splint--a necessary tool in the management of battlefield injuries**. *J R Army Med Corps* 2003, **149**(4):291-293.

171. Ellerton J, Milani M, Blancher M, Zen-Ruffinen G, Skaiaa SC, Brink B, Lohani A, Paal P: **Managing moderate and severe pain in mountain rescue**. *High Alt Med Biol* 2014, **15**(1):8-14.

172. Wedmore IS, Butler FK, Jr.: **Battlefield Analgesia in Tactical Combat Casualty Care**. *Wilderness Environ Med* 2017, **28**(2S):S109-S116.

173. Russell KW, Scaife CL, Weber DC, Windsor JS, Wheeler AR, Smith WR, Wedmore I, McIntosh SE, Lieberman JR, Wilderness Medical S: **Wilderness Medical Society practice guidelines for the treatment of acute pain in remote environments: 2014 update**. *Wilderness Environ Med* 2014, **25**(4 Suppl):S96-104.

174. Iserson KV: **An hypnotic suggestion: review of hypnosis for clinical emergency care**. *J Emerg Med* 2014, **46**(4):588-596.

175. Buttner B, Mansur A, Kalmbach M, Hinz J, Volk T, Szalai K, Roessler M, Bergmann I: **Prehospital ultrasound-guided nerve blocks improve reduction-feasibility of dislocated extremity injuries compared to systemic analgesia. A randomized controlled trial**. *PLoS One* 2018, **13**(7):e0199776.

176. Wu JJ, Lollo L, Grabinsky A: **Regional anesthesia in trauma medicine**. *Anesthesiol Res Pract* 2011, **2011**:713281.

177. Selbst SM, Fein JA: **Sedation and analgesia**. In: *Textbook of Pediatric Emergency Medicine.* 5th edn. Edited by Fleisher GR, Ludwig S, Henretig FM. Philadelphia: Lippincott Williams and Wilkins; 2006: 69.

178. Ireland S, Endacott R, Cameron P, Fitzgerald M, Paul E: **The incidence and significance of accidental hypothermia in major trauma--a prospective observational study**. *Resuscitation* 2011, **82**(3):300-306.

179. Trentzsch H, Huber-Wagner S, Hildebrand F, Kanz KG, Faist E, Piltz S, Lefering R, TraumaRegistry DGU: **Hypothermia for prediction of death in severely injured blunt trauma patients**. *Shock* 2012, **37**(2):131-139.

180. Strapazzon G, Procter E, Paal P, Brugger H: **Pre-hospital core temperature measurement in accidental and therapeutic hypothermia**. *High Alt Med Biol* 2014, **15**(2):104-111.

181. Pasquier M, Carron PN, Rodrigues A, Dami F, Frochaux V, Sartori C, Deslarzes T, Rousson V: **An evaluation of the Swiss staging model for hypothermia using hospital cases and case reports from the literature**. *Scand J Trauma Resusc Emerg Med* 2019, **27**(1):60.

182. Haverkamp FJC, Giesbrecht GG, Tan E: **The prehospital management of hypothermia - An up-to-date overview**. *Injury* 2018, **49**(2):149-164.

183. Henriksson O, Lundgren P, Kuklane K, Holmer I, Naredi P, Bjornstig U: **Protection against cold in prehospital care: evaporative heat loss reduction by wet clothing removal or the addition of a vapor barrier--a thermal manikin study**. *Prehosp Disaster Med* 2012, **27**(1):53-58.

184. Henriksson O, Lundgren PJ, Kuklane K, Holmer I, Giesbrecht GG, Naredi P, Bjornstig U: **Protection against cold in prehospital care: wet clothing removal or addition of a vapor barrier**. *Wilderness Environ Med* 2015, **26**(1):11-20.

185. Dow J, Giesbrecht GG, Danzl DF, Brugger H, Sagalyn EB, Walpoth B, Auerbach PS, McIntosh SE, Nemethy M, McDevitt M *et al*: **Wilderness Medical Society Clinical Practice Guidelines for the Out-of-Hospital Evaluation and Treatment of Accidental Hypothermia: 2019 Update**. *Wilderness Environ Med* 2019, **30**(4S):S47-S69.

186. Allen PB, Salyer SW, Dubick MA, Holcomb JB, Blackbourne LH: **Preventing hypothermia: comparison of current devices used by the US Army in an in vitro warmed fluid model**. *J Trauma* 2010, **69 Suppl 1**:S154-161.

187. Zasa M, Flowers N, Zideman D, Hodgetts TJ, Harris T: **A torso model comparison of temperature preservation devices for use in the prehospital environment**. *Emerg Med J* 2016, **33**(6):418-422.

188. Oliver SJ, Brierley JL, Raymond-Barker PC, Dolci A, Walsh NP: **Portable Prehospital Methods to Treat Near-Hypothermic Shivering Cold Casualties**. *Wilderness Environ Med* 2016, **27**(1):125-130.

189. Zafren K, Giesbrecht GG, Danzl DF, Brugger H, Sagalyn EB, Walpoth B, Weiss EA, Auerbach PS, McIntosh SE, Nemethy M *et al*: **Wilderness Medical Society practice guidelines for the out-of-hospital evaluation and treatment of accidental hypothermia: 2014 update**. *Wilderness Environ Med* 2014, **25**(4 Suppl):S66-85.

190. Brooks B, Deakin CD: **Relationship between oxygen concentration and temperature in an exothermic warming device**. *Emerg Med J* 2017, **34**(7):472-474.

191. Lehavi A, Yitzhak A, Jarassy R, Heizler R, Katz YS, Raz A: **Comparison of the performance of battery-operated fluid warmers**. *Emerg Med J* 2018, **35**(9):564-570.

192. Tomazin I, Ellerton J, Reisten O, Soteras I, Avbelj M, International Commission for Mountain Emergency M: **Medical standards for mountain rescue operations using helicopters: official consensus recommendations of the International Commission for Mountain Emergency Medicine (ICAR MEDCOM)**. *High Alt Med Biol* 2011, **12**(4):335-341.

193. Ausserer J, Moritz E, Stroehle M, Brugger H, Strapazzon G, Rauch S, Mair P, International Alpine Trauma Registry Study G: **Physician staffed helicopter emergency medical systems can provide advanced trauma life support in mountainous and remote areas**. *Injury* 2017, **48**(1):20-25.

194. Pasquier M, Geiser V, De Riedmatten M, Carron PN: **Helicopter rescue operations involving winching of an emergency physician**. *Injury* 2012, **43**(9):1377-1380.

195. Sun H, Samra NS, Kalakoti P, Sharma K, Patra DP, Dossani RH, Thakur JD, Disbrow EA, Phan K, Veeranki SP *et al*: **Impact of Prehospital Transportation on Survival in Skiers and Snowboarders with Traumatic Brain Injury**. *World Neurosurg* 2017, **104**:909-918 e908.

196. de Jongh MA, van Stel HF, Schrijvers AJ, Leenen LP, Verhofstad MH: **The effect of Helicopter Emergency Medical Services on trauma patient mortality in the Netherlands**. *Injury* 2012, **43**(9):1362-1367.

197. Galvagno SM, Jr., Haut ER, Zafar SN, Millin MG, Efron DT, Koenig GJ, Jr., Baker SP, Bowman SM, Pronovost PJ, Haider AH: **Association between helicopter vs ground emergency medical services and survival for adults with major trauma**. *JAMA* 2012, **307**(15):1602-1610.

198. Bekelis K, Missios S, Mackenzie TA: **Prehospital helicopter transport and survival of patients with traumatic brain injury**. *Ann Surg* 2015, **261**(3):579-585.

199. Andruszkow H, Lefering R, Frink M, Mommsen P, Zeckey C, Rahe K, Krettek C, Hildebrand F: **Survival benefit of helicopter emergency medical services compared to ground emergency medical services in traumatized patients**. *Crit Care* 2013, **17**(3):R124.

200. Zhu TH, Hollister L, Opoku D, Galvagno SM, Jr.: **Improved Survival for Rural Trauma Patients Transported by Helicopter to a Verified Trauma Center: A Propensity Score Analysis**. *Acad Emerg Med* 2018, **25**(1):44-53.

201. Stengel D, Rademacher G, Ekkernkamp A, Guthoff C, Mutze S: **Emergency ultrasound-based algorithms for diagnosing blunt abdominal trauma**. *Cochrane Database Syst Rev* 2015(9):CD004446.

202. Price DD, Wilson SR, Murphy TG: **Trauma ultrasound feasibility during helicopter transport**. *Air Med J* 2000, **19**(4):144-146.

203. Snaith B, Hardy M, Walker A: **Emergency ultrasound in the prehospital setting: the impact of environment on examination outcomes**. *Emerg Med J* 2011, **28**(12):1063-1065.

204. Kirkpatrick AW, Sirois M, Laupland KB, Liu D, Rowan K, Ball CG, Hameed SM, Brown R, Simons R, Dulchavsky SA *et al*: **Hand-held thoracic sonography for detecting post-traumatic pneumothoraces: the Extended Focused Assessment with Sonography for Trauma (EFAST)**. *J Trauma* 2004, **57**(2):288-295.

205. Press GM, Miller SK, Hassan IA, Alade KH, Camp E, Junco DD, Holcomb JB: **Prospective evaluation of prehospital trauma ultrasound during aeromedical transport**. *J Emerg Med* 2014, **47**(6):638-645.

206. Ketelaars R, Hoogerwerf N, Scheffer GJ: **Prehospital chest ultrasound by a dutch helicopter emergency medical service**. *J Emerg Med* 2013, **44**(4):811-817.

207. Gottlieb M, Holladay D, Peksa GD: **Ultrasonography for the Confirmation of Endotracheal Tube Intubation: A Systematic Review and Meta-Analysis**. *Ann Emerg Med* 2018, **72**(6):627-636.

208. You-Ten KE, Siddiqui N, Teoh WH, Kristensen MS: **Point-of-care ultrasound (POCUS) of the upper airway**. *Can J Anaesth* 2018, **65**(4):473-484.

209. Siddiqui N, Yu E, Boulis S, You-Ten KE: **Ultrasound Is Superior to Palpation in Identifying the Cricothyroid Membrane in Subjects with Poorly Defined Neck Landmarks: A Randomized Clinical Trial**. *Anesthesiology* 2018, **129**(6):1132-1139.

210. Quick JA, Uhlich RM, Ahmad S, Barnes SL, Coughenour JP: **In-flight ultrasound identification of pneumothorax**. *Emerg Radiol* 2016, **23**(1):3-7.

211. Peters J, Ketelaars R, van Wageningen B, Biert J, Hoogerwerf N: **Prehospital thoracostomy in patients with traumatic circulatory arrest: results from a physician-staffed Helicopter Emergency Medical Service**. *Eur J Emerg Med* 2017, **24**(2):96-100.

212. Kearns MJ, Walley KR: **Tamponade: Hemodynamic and Echocardiographic Diagnosis**. *Chest* 2018, **153**(5):1266-1275.

213. Bodson L, Bouferrache K, Vieillard-Baron A: **Cardiac tamponade**. *Curr Opin Crit Care* 2011, **17**(5):416-424.

214. Schairer JR, Biswas S, Keteyian SJ, Ananthasubramaniam K: **A systematic approach to evaluation of pericardial effusion and cardiac tamponade**. *Cardiol Rev* 2011, **19**(5):233-238.

215. Co SJ, Yong-Hing CJ, Galea-Soler S, Ruzsics B, Schoepf UJ, Ajlan A, Farand P, Nicolaou S: **Role of imaging in penetrating and blunt traumatic injury to the heart**. *Radiographics* 2011, **31**(4):E101-115.

216. Huang YK, Lu MS, Liu KS, Liu EH, Chu JJ, Tsai FC, Lin PJ: **Traumatic pericardial effusion: impact of diagnostic and surgical approaches**. *Resuscitation* 2010, **81**(12):1682-1686.

217. Brun PM, Bessereau J, Levy D, Billeres X, Fournier N, Kerbaul F: **Prehospital ultrasound thoracic examination to improve decision making, triage, and care in blunt trauma**. *Am J Emerg Med* 2014, **32**(7):817 e811-812.

218. McNeil CR, McManus J, Mehta S: **The accuracy of portable ultrasonography to diagnose fractures in an austere environment**. *Prehosp Emerg Care* 2009, **13**(1):50-52.

219. Cho KH, Lee SM, Lee YH, Suh KJ: **Ultrasound diagnosis of either an occult or missed fracture of an extremity in pediatric-aged children**. *Korean J Radiol* 2010, **11**(1):84-94.

220. Robba C, Cardim D, Tajsic T, Pietersen J, Bulman M, Rasulo F, Bertuetti R, Donnelly J, Xiuyun L, Czosnyka Z *et al*: **Non-invasive Intracranial Pressure Assessment in Brain Injured Patients Using Ultrasound-Based Methods**. *Acta Neurochir Suppl* 2018, **126**:69-73.

221. O'Dochartaigh D, Douma M: **Prehospital ultrasound of the abdomen and thorax changes trauma patient management: A systematic review**. *Injury* 2015, **46**(11):2093-2102.

222. Zanobetti M, Coppa A, Nazerian P, Grifoni S, Scorpiniti M, Innocenti F, Conti A, Bigiarini S, Gualtieri S, Casula C *et al*: **Chest Abdominal-Focused Assessment Sonography for Trauma during the primary survey in the Emergency Department: the CA-FAST protocol**. *Eur J Trauma Emerg Surg* 2018, **44**(6):805-810.

223. Jorgensen H, Jensen CH, Dirks J: **Does prehospital ultrasound improve treatment of the trauma patient? A systematic review**. *Eur J Emerg Med* 2010, **17**(5):249-253.

224. Anonymous: **BET 2: Is prehospital focused abdominal ultrasound useful during triage at mass casualty incidents?** *Emerg Med J* 2013, **30**(7):596-597.

225. Fragou M, Gravvanis A, Dimitriou V, Papalois A, Kouraklis G, Karabinis A, Saranteas T, Poularas J, Papanikolaou J, Davlouros P *et al*: **Real-time ultrasound-guided subclavian vein cannulation versus the landmark method in critical care patients: a prospective randomized study**. *Crit Care Med* 2011, **39**(7):1607-1612.

226. Ketelaars R, Stollman JT, van Eeten E, Eikendal T, Bruhn J, van Geffen GJ: **Emergency physician-performed ultrasound-guided nerve blocks in proximal femoral fractures provide safe and effective pain relief: a prospective observational study in The Netherlands**. *Int J Emerg Med* 2018, **11**(1):12.

227. van Kraaij DJ, Hovestad-Witterland AH, de Metz M, Vollaard EJ: **A comparison of the effects of nabumetone vs meloxicam on serum thromboxane B2 and platelet function in healthy volunteers**. *Br J Clin Pharmacol* 2002, **53**(6):644-647.

228. Marland S, Ellerton J, Andolfatto G, Strapazzon G, Thomassen O, Brandner B, Weatherall A, Paal P: **Ketamine: use in anesthesia**. *CNS Neurosci Ther* 2013, **19**(6):381-389.

229. Paix BR, Capps R, Neumeister G, Semple T: **Anaesthesia in a disaster zone: a report on the experience of an Australian medical team in Banda Aceh following the 'Boxing Day Tsunami'**. *Anaesth Intensive Care* 2005, **33**(5):629-634.

230. Karlow N, Schlaepfer CH, Stoll CRT, Doering M, Carpenter CR, Colditz GA, Motov S, Miller J, Schwarz ES: **A Systematic Review and Meta-analysis of Ketamine as an Alternative to Opioids for Acute Pain in the Emergency Department**. *Acad Emerg Med* 2018, **25**(10):1086-1097.

231. Todd KH: **A Review of Current and Emerging Approaches to Pain Management in the Emergency Department**. *Pain Ther* 2017, **6**(2):193-202.

232. Jephcott C, Grummet J, Nguyen N, Spruyt O: **A review of the safety and efficacy of inhaled methoxyflurane as an analgesic for outpatient procedures**. *Br J Anaesth* 2018, **120**(5):1040-1048.

233. Wilkes M, Heath EC, Mason NP: **Methoxyflurane for Procedural Analgesia at 4470 m Altitude**. *Wilderness Environ Med* 2018, **29**(3):388-391.

234. Wakai A, O'Sullivan R, McCabe A: **Intra-articular lignocaine versus intravenous analgesia with or without sedation for manual reduction of acute anterior shoulder dislocation in adults**. *Cochrane Database Syst Rev* 2011(4):CD004919.

235. Kelly Y, Yonga J: **Regional anesthesia of the thorax and extremities.** In: *Roberts and Hedges’ Clinical Procedures in Emergency Medicine and Acute Care,.* 7th edn. Edited by Roberts J, Hedges J. Amsterdam, NL: Elsevier; 2019: 560-587.

236. Latifzai K, Sites BD, Koval KJ: **Orthopaedic anesthesia - part 2. Common techniques of regional anesthesia in orthopaedics**. *Bull NYU Hosp Jt Dis* 2008, **66**(4):306-316.

237. Gadsden J, Warlick A: **Regional anesthesia for the trauma patient: improving patient outcomes**. *Local Reg Anesth* 2015, **8**:45-55.

238. Kelly J, Younga J: **Regional anesthesia of the thorax and extremities**. In: *Roberts and Hedges’ Clinical Procedures in Emergency Medicine and Acute Care.* edn. Edited by Roberts J, Hedges J. Amsterdam, NL: Elsevier; 2019: 560-587.

239. Hards M, Brewer A, Bessant G, Lahiri S: **Efficacy of Prehospital Analgesia with Fascia Iliaca Compartment Block for Femoral Bone Fractures: A Systematic Review**. *Prehosp Disaster Med* 2018, **33**(3):299-307.

240. Skaiaa SC, Brattebo G, Assmus J, Thomassen O: **The impact of environmental factors in pre-hospital thermistor-based tympanic temperature measurement: a pilot field study**. *Scand J Trauma Resusc Emerg Med* 2015, **23**:72.
